# Scale Space Calibrates Present and Subsequent Spatial Learning in Barnes Maze in Mice

**DOI:** 10.1523/ENEURO.0505-22.2023

**Published:** 2023-06-02

**Authors:** Yuto Tachiki, Yusuke Suzuki, Mutsumi Kurahashi, Keisuke Oki, Özgün Mavuk, Takuma Nakagawa, Shogo Ishihara, Yuichiro Gyoten, Akira Yamamoto, Itaru Imayoshi

**Affiliations:** 1 Laboratory of Brain Development and Regeneration, Division of Systemic Life Science, Kyoto University Graduate School of Biostudies, Kyoto, 606-8501, Japan; 2 Center for Living Systems Information Science, Kyoto University Graduate School of Biostudies, Kyoto, 606-8501, Japan; ^3^Institute for Frontier Life and Medical Sciences, Laboratory of Deconstruction of Stem Cells, Kyoto University, Kyoto, 606-8501, Japan

**Keywords:** Barnes maze, mice, scale space, spatial learning, spatial navigation, spatial representation

## Abstract

Animals are capable of representing different scale spaces from smaller to larger ones. However, most laboratory animals live their life in a narrow range of scale spaces like homecages and experimental setups, making it hard to extrapolate the spatial representation and learning process in large scale spaces from those in conventional scale spaces. Here, we developed a 3-m diameter Barnes maze (BM3), then explored whether spatial learning in the Barnes maze (BM) is calibrated by scale spaces. Spatial learning in the BM3 was successfully established with a lower learning rate than that in a conventional 1-m diameter Barnes maze (BM1). Specifically, analysis of exploration strategies revealed that the mice in the BM3 persistently searched certain places throughout the learning, while such places were rapidly decreased in the BM1. These results suggest dedicated exploration strategies requiring more trial-and-errors and computational resources in the BM3 than in the BM1, leading to a divergence of spatial learning between the BM1 and the BM3. We then explored whether prior learning in one BM scale calibrates subsequent spatial learning in another BM scale, and found asymmetric facilitation such that the prior learning in the BM3 facilitated the subsequent BM1 learning, but not vice versa. Thus, scale space calibrates both the present and subsequent BM learning. This is the first study to demonstrate scale-dependent spatial learning in BM in mice. The couple of the BM1 and the BM3 would be a suitable system to seek how animals represent different scale spaces with underlying neural implementation.

## Significance Statement

Animals are capable of representing different scale spaces. However, whether scale space calibrates goal-directed spatial learning remains unclear. The Barnes maze (BM) is a well-established experimental paradigm to evaluate spatial learning in rodents. Here, we developed a larger scale 3-m diameter Barnes maze (BM3) then compared various navigation features in mice between the BM3 and a conventional 1-m diameter Barnes maze (BM1). We demonstrated that spatial learning on the BM3 was established, but required more trial-and-error and computational resources than in the BM1, prompting mice to visit certain places persistently. Such learning experiences in the BM3 facilitated subsequent spatial learning in the BM1, but not vice versa. These results suggest that scale space calibrates present and subsequent spatial learning.

## Introduction

Animals including humans are capable of representing different scale spaces from smaller to larger ones. For example, bats and wild rodents can navigate from the order of centimeters of detail in the vicinity of burrows and feeding grounds, to the order of kilometers between their burrow and feeding grounds (Geva-Sagiv et al., 2015). Understanding how animals acquire spatial representations over different scale spaces and how to incorporate them into a cognitive map are close to the nature of spatial learning. However, most laboratory animals live their life in a narrow range of scale space, between homecages and experimental setups, consequently it is difficult to extrapolate the spatial representation and learning process in large scale spaces from those in conventional scale spaces. Hence, examining spatial learning in both conventional and large scale spaces has become essential.

Several types of neurons act as a unit interactively accounting for a cognitive map (Knierim et al., 1998). For example, hippocampal place cells encode one or more places termed place fields where they fired with the spatial resolution of dozens of centimeters order when rodents were located in the places (O’Keefe and Conway, 1978). Grid cells in the medial entorhinal cortex are active when the animal passes the apex of a hexagonal grid over its environment (Hafting et al., 2005). Compared with place cells, spatial resolution of grid cells widely ranges from about dozens of centimeters to several meters (Brun et al., 2008).

However, it is unlikely that any scale spaces are represented at a single, common spatial resolution; if one was to try to perform navigation on the order of kilometers with place cells of centimeter resolution, the number of required spatially selective neurons would be larger than the total number of neurons in a brain. Rather, spatial resolution would be different per scale space to be represented, or each place cell would have multiple or enlarged place fields (Geva-Sagiv et al., 2015). Several studies demonstrated that spatial scale in the environment controls the number and the size of place fields of place cells Muller and Kubie, 1987; O’Keefe and Burgess, 1996; Fenton et al., 2008; (Kjelstrup et al., 2008; Park et al., 2011; Rich et al., 2014; Harland et al., 2021; Sarel et al., 2022). Although these studies suggested the transiently existing scale-dependent spatial representation, its learning and memory processes have not been determined with the lack of suitable experimental systems or paradigms.

The Barnes maze (BM) test was originally developed by Carol A. Barnes in 1979 (Barnes, 1979), and nowadays is one of well-established experimental paradigms to test spatial learning and memory in rodents. Briefly, a mouse was released in a bright, circular open field with 12 holes equally spaced along with the edge of the field. One escape box is attached under any one of the holes. The mouse can escape from the field by entering the escape box, which is the “goal” in this maze. Because the location of the goal is fixed per mouse, it can optimize navigation from start to goal, by updating spatial representation of the maze across repeated training. It is well documented that the BM learning depends on the hippocampus, as a number of studies reported that hippocampal lesioned rodents exhibited impaired spatial memory and navigation in the BM task (Bach et al., 1995; Mayford et al., 1996; Raber et al., 2004). Conventional Barnes maze test is performed on a field varying from 70 to 130 cm in diameter (Bach et al., 1995; Raber et al., 2004; Rosenfeld and Ferguson, 2014; Pitts, 2018), and no studies have compared spatial learning between the Barnes mazes of different scale spaces.

Here, we developed a 3-m diameter Barnes maze (BM3) which is three times larger than a conventional 1-m diameter Barnes maze (BM1). Comparing a variety of behavioral features in mice in the BM3 with those in the BM1, we examined whether spatial learning in the immediate BM is calibrated by scale spaces. Furthermore, it has been widely accepted that animals learn not only solutions of an immediate task per se, but also how to find the solutions (learning to learn; Harlow, 1949). This meta-learning process leads to few-shot learning in future tasks that are variants of the previously learned task (for review, see Wang, 2021). To explore whether the meta-learning process is also calibrated by scale spaces, facilitation effects from prior learning in one BM scale (e.g., BM3) to subsequent spatial learning in the another BM scale (e.g., BM1) were evaluated.

## Materials and Methods

### Animal

A total of 115 naive male C57BL/6J mice (Japan SLC) were used in this study. All mice were two to three months old on the first day of the initial task. The mice were group-housed in a standard laboratory environment, maintained on a 12/12 h light/dark cycle at 23–24°C temperature and 40% – 50% relative humidity. Food (pellets; Japan SLC) and water were provided *ad libitum*. The present behavioral testing was done during the dark phase. After the behavioral experiments, all mice were killed by cervical dislocation. All animal procedures were performed in accordance with the Kyoto University animal care committee’s regulations (permit numbers: Lif-K22008).

### Cohort

**Figure 1.**
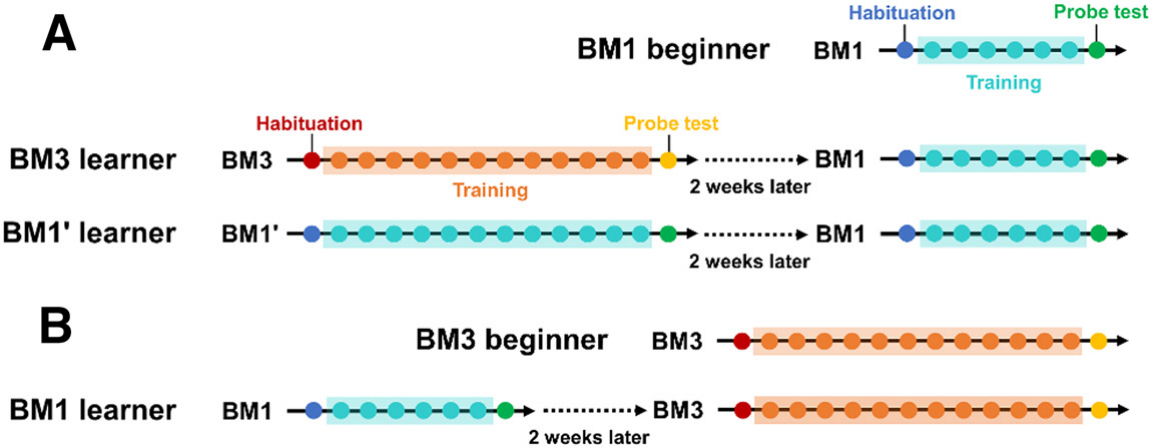
Experimental schedules for the BM beginners and learners. Experimental schedules for the BM1 beginner, the BM3 learner and the BM1' learner (***A***), and those for the BM3 beginner and the BM1 learner (***B***). Each small circle represents a day. Each color of each circle represents an experimental phase of either BM experiment; blue, cyan, and green express the habituation, training, and probe test in the BM1 and BM1', respectively, while red, orange, and yellow are the habituation, training, and probe test in the BM3, respectively.

Each mouse was assigned to one of seven cohorts, depending on the history of tasks that they were engaged in Table 1. Cohort 1 was engaged only in the 1-m diameter maze (BM1). Cohort 2 was engaged only in the 3-m diameter maze (BM3). Cohorts 3–5 were engaged in the two tasks with the blank of 14 d between the tasks. Cohort three was engaged in the BM3 at the first task, then the BM1 at the second task. Cohort 4 was engaged in the modified BM1 (BM1') at the first task, then the BM1 task at the second task; the BM1' task was the same as the BM1 task, except that the spatial cues were replaced by new independent ones, and that the training lasted for 12 d as much as that in the BM3. Thus, Cohort 4 and Cohort 5 learned the prior BM3 task and the BM1' task for 12 d, respectively, and thereby, they were treated as the BM3 learner and the BM1' learner, respectively (Fig. 1*A*). Comparing between the two, we explored whether spatial learning is facilitated by prior learning in a large scale space more than that in a small scale space given the same training days. Cohort 5 was engaged in the BM3 then the BM1, and termed as the BM1 learner (Fig. 1*B*). Cohort S1 was subjected to check whether exploration patterns under scopolamine treatment in the probe test in the BM1 as previously reported (Suzuki and Imayoshi, 2017) are maintained even in the BM3. The instance of Cohort S1 was the same with that in the Cohort 2 except that scopolamine hydrobromide (Santa Cruz Biotechnology) was injected intraperitoneally 20 min before starting the probe test, where the scopolamine was prepared as a 0.3 mg/ml stock solution in 0.9% saline, so as to be 3 mg of scopolamine hydrobromide/kg of body weight. Cohort S2 was engaged in the contextual fear conditioning (CFC) at the first task, then the BM1 at the second task, with 30-d blank between the tasks, to check whether prior learning different from the BM task does not facilitate spatial learning in the subsequent BM1 task.

**Table 1:** Cohort and task instance

Cohort	*N*	Age (month old)	Sex	Instance 1	Instance 2
1	20	2–3	Male	BM1	
2	20	2	Male	BM3	
3	20	3	Male	BM3	BM1
4	17	2	Male	BM1'	BM1
5	14^*a*^	3	Male	BM1	BM3
S1	16	2	Male	BM3^*b*^	
S2	8	2	Male	CFC	BM1

*N* = number of mice in each cohort at instance 1. CFC = contextual fear conditioning.

a One mouse was found dead in a blank between instances 1 and 2.

b Scopolamine hydrobromide was injected 20 min before the beginning of the probe test.

### The 1-m diameter Barnes maze (BM1)

#### Apparatus

**Figure 2.**
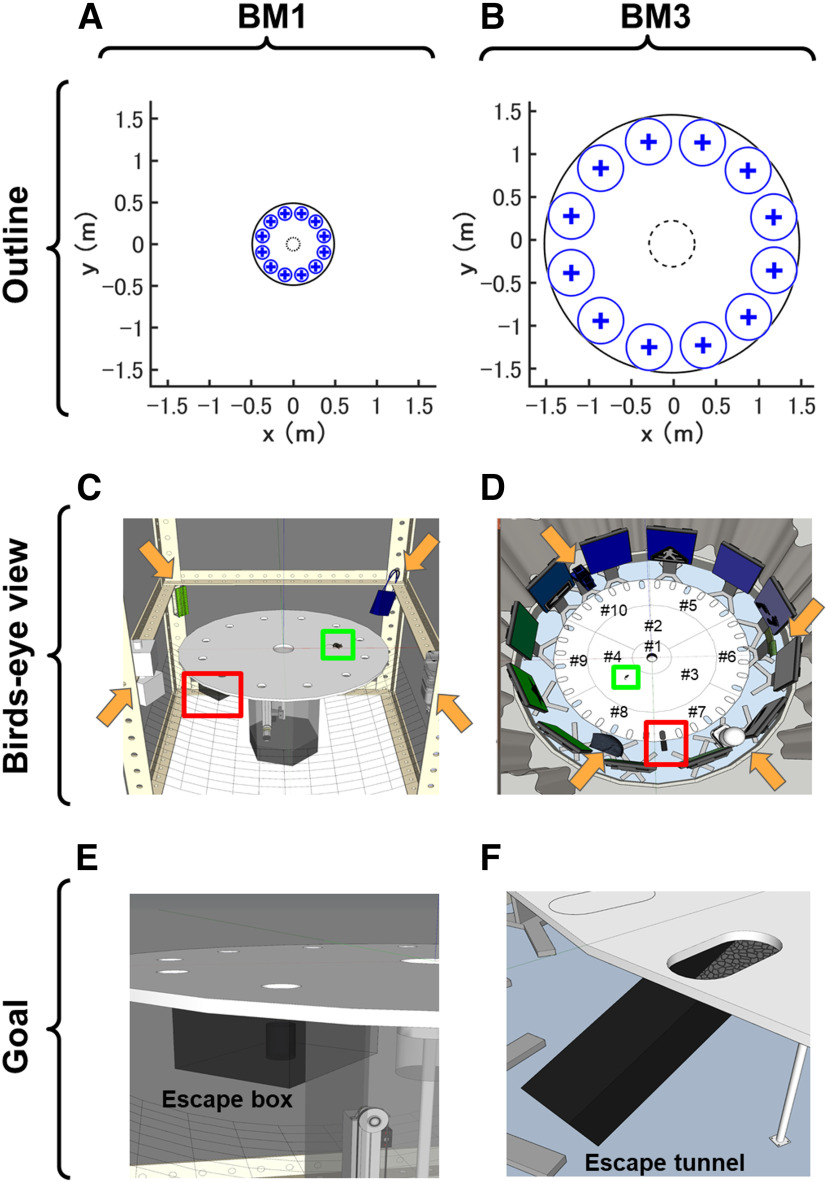
Architecture of the conventional 1-m diameter Barnes maze (BM1) and the 3-m diameter Barnes maze (BM3). ***A–F***, The architecture of BM1 and BM3. Top, middle, and bottom rows indicate outlines (***A***, ***B***), birds-eye views (***C***, ***D***), and enlarged images of goals (***E***, ***F***), respectively. In the outline, the display unit of vertical (*y*) and horizontal (*x*) axes in meters. The largest circle drawn by black solid line represents the edge of each maze. The “+” markers and blue-line circles around them represent the goal or dummy holes and the areas where hole visiting was scored, respectively. The radius of each area was set at ∼80 mm for the BM1 (***A***) and 270 mm for the BM3 (***B***). While these blue areas were designed so that these sizes in the BM3 were the triple of those in the BM1, the radius of the holes was ∼40 mm larger in the BM3 than in the BM1 (see Materials and Methods); therefore, we set the radius of the target fields in the BM3 to be the sum of the “margin” and exactly triple of the radius of the target field in the BM1. Namely, the number of errors was determined by the number that mice entered the blue-line circled areas until they goaled. Likewise, in the analysis of the probe test of spatial memory, time spent around each hole was the duration that mice stayed in the blue-line circled area. The black dashed-line circles represent the start areas with a lift transporting mice to the field. In strategy analysis, trajectory data while mice stayed in the start area since they entered the field were discarded, so that initial wobbly trajectory around the lift did not affect the classification of navigation strategy (see details in Materials and Methods). ***C***, ***D***, In the birds-eye views, the orange arrows point to the locations of distal spatial cues. The red and green squares represent escape box/tunnel and mice, respectively. Each number (#) in ***D*** corresponds to each component of the BM3 arena. Escape box and tunnel were zoomed in ***E***, ***F***, respectively.

The BM1 task was conducted on a custom-made Barnes maze system (Bio-Medica; Fig. 2*A*,*C*,*E*). Through the task, the mice learned to elaborate cognitive maps of the maze, and to take efficient navigation from the start to the goal on the maze. The maze was composed of a circular open arena with 98 cm in diameter and 72 cm in height from the floor. A start-lift was set at the center of the arena surface. The scaffold of the lift (10 cm in diameter) was made of the same material as the arena and was held 20 cm below the surface of the arena before the initiation of each trial. At the start of each trial, the lift transported a mouse to the center of the arena surface. The vertical movement of the lift was programmed so that the experimenter could control it externally at any time. Twelve holes were equally spaced around the perimeter at a distance of 40 cm from the centroid where the diameter of each hole was 4 cm. A black iron escape box (17 × 14 × 7 cm), which had paper cage bedding on its bottom, was located under one of the holes (Fig. 2*E*). This hole is the goal of this navigation task, and the remaining holes were considered as distractors. The location of the goal was consistent for a given mouse, but was randomized across mice. The entire apparatus was set within a cube-shaped outer enclosure (130 × 130 × 180 cm) with black curtains to obscure the outside scene of the arena and absorb background noise. One white color projection LED light was mounted on the center of the ceiling of the enclosure to ensure uniform and intense illumination of the arena (600 lx on the arena surface). Unique 3D object as a spatial cue was set at each of four corners of the enclosure at a height of 86 cm from the floor.

Behavior during the trials was recorded using a GigE Vision camera (UI-5240SE-NIR; IDS Imaging Development Systems GmbH). The camera was mounted on the ceiling of the enclosure (93.5 cm above the maze center), which could achieve a spatial resolution of 1.96 mm/pixel. Each image frame (500 × 500 pixels, with 2× binning) was acquired at the rising edge on a 20-Hz pulse counter and displayed on a monitor outside of the enclosure. All programs used for data acquisition, processing, saving, and synchronized device controls, were written in LabVIEW 2013 (National Instruments).

#### Experimental procedure

The protocol of the BM1 task consists of three phases: habituation on day 0, training from day 1 to 6, and the probe test on day 7. In all phases, mice were moved from the breeding rack to the experimental room 30 min before the experiment, and stayed in their homecages on a standby-rack in the experimental room while they were waiting for each trial. Immediately before the trial, the mouse was moved from the cage to the bottom of the lift via an opaque acrylic cylinder. At the start of the trial, the mouse was lifted up to the arena surface simultaneously with the start of recording. After every trial, the maze was thoroughly cleaned with 70% ethanol solution and dried. In the habituation phase, mice were allowed to freely explore the arena for 5 min. Afterwards they were moved to the escape box for 5 min, then they were returned to the homecage. This procedure was executed once per mouse. In the training phase, mice explored the arena until the mouse had successfully entered the escape box within 10 min. When the experimenter confirmed it, the recording was turned off, then brought the mouse back to the homecage. Otherwise, the experimenter picked up the mouse manually and returned it to the homecage by the cylinder. Trial was incremented by 1 if all mice completed the current trial, and three trials were performed in each training day. Twenty-four hours after the last day of training, mice were subjected to the probe test. The procedure of the probe test was identical to that of the habituation except that they were moved to the homecage without staying in the escape box after the trial ended. During the probe trial, the mice explored the field in the absence of the escape box for 5 min. If mice acquired spatial memory of the goal, they would focally search around the hole where the escape box was located in the training.

### The 3-m diameter Barnes maze (BM3)

#### Apparatus

The dedicated system for the BM3 was custom-built (Bio-Medica; Fig. 2*B*,*D*,*F*). The material of the circular arena was the same as the BM1, whereas the diameter was extended to 300 cm. The arena was constituted from 10 parts (#1–10; Fig. 2*D*), and these parts were seamlessly connected to generate a 3-m diameter circular arena. A start-lift was set at the center of the arena surface. The lift was made of the same material as the arena, and the diameter was 11.6 cm. This lift vertically transported each mouse to the arena surface (79 cm in height from the floor) from lower end (22 cm below the arena surface) at each trial start. Thirty-six slotted holes were equally spaced along with the parts #5–10 which are the circumference parts of the arena. The width, length, radius of each hole were 8, 16, and 4 cm, respectively. In this study, twenty-four holes were closed by lids of the same material as the arena surface, while every three holes (= 12 holes) were opened. As in the BM1 task, each goal hole for each mouse was pseudo-randomly chosen, and an acrylic escape tunnel (width, height, and length were 9, 8.5, and 30.5 cm, respectively) was connected under the goal hole at a gentle slope, 20°, so that the mice can easily run into the tunnel from the arena. The floor of the escape tunnel was covered with paper cage bedding (Fig. 2*F*). A dummy tunnel was connected to the hole opposite to the goal hole. The design of the dummy tunnel is the same as the escape tunnel except that it was floorless, so that mice cannot escape. We expect that the mice taking learning-irrelevant strategies such as reaching the escape tunnel by exclusively seeking along the edge of the arena could be easily identified, because such mice would be distracted by the dummy tunnel. Twelve displays (112.5 × 65.5 cm; 3840 × 2160 pixel; DME-4K50D; DMM.com) were arranged so that these surrounded the arena at equal space. Each display presented 1 unique color, and every two displays presented a unique graphic. Four unique 3D objects were placed between the arena and the displays at equal space. Thus, the total 16 objects were presented as unique spatial cues. To mask environmental sounds that might allow sound localization, white noise was played from the speakers of all displays during the task. The loudness level was 55 dB in the arena. Eight room lights attached at the ceiling uniformly illuminated the arena at 500 lx. To mask scene and sound outside the arena, the BM3 apparatus described above were located in a compartment that was separated by a white round curtain.

Behavior during the trials was recorded using a GigE Vision camera (UI-5220SE; IDS Imaging Development Systems GmbH). The camera was mounted on the ceiling of the compartment (160 cm above the maze center) and was loaded with the ultrawide angle lens (Theia MY125M, Nittoh Inc.), such that a spatial resolution achieved 7.43 mm/pixel. Each image frame (404 × 404 pixels, with 2× binning) was acquired at 20 Hz and displayed on a monitor outside of the compartment. All programs used for data acquisition, processing, saving, and synchronized device controls, were written in LabVIEW 2017 (National Instruments).

#### Experimental procedure

The protocol of the BM3 consists of habituation (day 0), training (days 1–12), and the probe test (day 13). Immediately before the trial, the experimenter moved a mouse from the homecage to one of three releasing points equally spaced around the arena, by a long stainless ladle covered by a lid. Then, the experimenter released the mouse from the ladle into the bottom of the lift. During this move, the mice could not see outside, as the view was obscured by the lid on the ladle. Because equilibrioceptive and proprioceptive cues might reveal certain directions, the releasing points were pseudo-randomized across trials, but common within trials. Other procedures in the habituation, training and probe test were the same with those in the BM1, except that the escape tunnel was used instead of the escape box.

#### Contextual fear conditioning

The contextual fear conditioning test for Cohort S2 was performed for successive 2 d on the fear conditioning test system (O’HARA & CO., LTD). On day 1, the mice learned the association between the context and electric footshock (acquisition). The experimenter wearing a white lab coat moved mice homecage from the breeding rack to the standby rack in the experimental room, 30 min before the trial started. Immediately before the trial started, the experimenter moved each mouse from the homecage to the chamber by using the delivery cage filled by woodchip. The acquisition chamber was composed of an acrylic cube (17 × 15 × 12 cm), with a wall of black and white stripes. The trial was started after the mice entered the chamber, and lasted for 9 min. At 148 s from the trial start, an electric foot-shock (0.4 mA) was given from the grid floor for 2 s in duration, and was repeated five times with 90-s interval. After the trial ended, the mice were returned to the homecage via the delivery cage. The soundproof box, chamber, and the grid floor were cleaned with 70% ethanol before each trial started. The brightness in the chamber and experimental room was 70 and 80 lx, respectively.

Twenty-four hours after the acquisition, the mice were exposed to a novel context to confirm that the generalization of the fear memory did not occur (retrieval 1). In this context, the soundproof box and the chamber was cleaned with 70% propanol. The retrieval chamber was composed of an acrylic white cube (18 × 11 × 10.5 cm) paved by latex sheets. The room light was turned off, while the concealed light was turned on, such that the brightness in the chamber and the experimental room was 40 and 8 lx, respectively. The experimenter moved each mouse directly to the retrieval chamber from the homecage in the breeding rack, then trial was started. Each trial lasted for 6 min, and no foot-shocks were given. After the trial ended, the experimenter returned each mouse directly to the homecage from the chamber. Two hours after retrieval 1, the mice were re-exposed to the acquisition context (retrieval 2). The environment and procedure were the same as in the acquisition, except that the duration per trial was 6 min, and no foot-shocks were presented.

All behaviors in the chamber were recorded by a camera at 2 Hz. Freezing response as a conditioned response in each trial was detected when the number of pixels whose intensity changed between successive two frames were fewer than 30, and this state continued for >2 s. Freezing rate was calculated as a percentage of total duration of freezing response to total recording time for each mouse in each trial. We assume that contextual fear memory is formed if the freezing rate in the retrieval 2 was significantly higher than that in the retrieval 1.

### Data analysis

In the BM tasks, the mouse position coordinates for every recorded frame were estimated by either a shape adaptive mean shift or an ellipse detection algorithm implemented by the LabVIEW programs. The sequence of coordinates was transformed so that the target holes were located at the same points across mice. Conventional, strategy, and network analyses were then performed to extract the corresponding behavioral features (Extended Data Fig. 3-1). The definitions of these features are based on a previous study (Suzuki and Imayoshi, 2017).

10.1523/ENEURO.0505-22.2023.ext1 Extended Data 1 Codes for data acquisition and analysis. Download Extended Data 1, ZIP file.

Briefly, the conventional analysis calculated the number of errors, time of latency, travel distance to reach the target hole for each mouse in each trial in the training phase. The number of errors was the sum of the number of visits to the areas around the nontarget holes, where the radius of each area was set at ∼80 mm for the BM1 and 270 mm for the BM3 (Fig. 2*A*,*B*). The average of each feature across the three trials per day was calculated for each mouse. Learning curves of the BM1 and BM3 across trials were quantified by intercept and slope estimated from nonlinear exponential curve fitting. In the probe test, we calculated time spent around each hole, as the total duration that a mouse stayed at an area within a radius of each hole, where the radius was the same as above. As too long duration may prompt mice to explore holes other than the target hole while too short duration may contain limited information to determine whether spatial learning is established, we used the data for the first half (150 s) of each probe trial (300 s in total) based on our pilot tests.

The strategy analysis was conducted to find dynamic components of spatial learning. Trajectory in each trial were categorized as any one of spatial, serial, or random strategy by algorithm-based classification. In brief, if a mouse in a trial moved directly toward the target hole and the number of visits to dummy holes was less than three, the strategy was classified as “spatial.” The “serial” strategy was assigned when the mouse sequentially approached neighboring holes until they reached the target with fewer than three quadrant crossings. All remaining behavioral patterns were assigned the “random” strategy. For quantitative definition of each strategy, see Suzuki and Imayoshi (2017). The strategy analysis was applied to the data only in the training phase. To avoid overestimation such that the random strategy was assigned even to micromovements around the center of the arena, the trajectory data while the mice stayed within 8-cm (BM1) or 27-cm (BM3) distance of the center lift were discarded from the strategy analysis.

The network analysis for spatial navigation behavior in rodents was initially established by Weiss et al. (2012) demonstrated that the object exploration behaviors of rats could be visualized as structured networks of interconnected nodes. If it was applied for the Barnes maze task, simplifying network structures in navigation behaviors across spatial learning could be observed (Suzuki and Imayoshi, 2017). Briefly, a network is constituted from nodes and links between them. If a trajectory during navigation in an environment is expressed as a network, nodes and links can correspond to the places where certain behavior was observed, and transitions between the places, respectively. A node was generated at the coordinate clustering stopping points. When the traveling distance at least 20 successive frames was less than the threshold, one stopping coordinate was generated at the centroid of the points during the frames. The threshold was set at 4 cm, which was approximately half of the average mouse’s body length. Then, the City Clustering Algorithm recursively generated nodes using the following three steps until a convergence condition was satisfied (Rozenfeld et al., 2008; Weiss et al., 2012). First, the coordinates of a stopping point were selected and then integrated those coordinates into the nearest node when the distance between the stopping coordinates and the node was <4 cm. If a stop was not integrated into any nodes, the stop was treated as a new node. Second, the coordinates of all nodes were calculated, where each node was represented by the centroid of its constituent stops. Third, if all stopping coordinates were integrated into a node, the processing was stopped, otherwise returned to the first step.

A structure of a network can be quantified by a set of measures. The order was the number of nodes in the network. The degree measured the number of links connected to a node in the network. The density is the ratio of the number of links that are actually present in a network to the number of links that are theoretically possible in the network. The clustering coefficient is the probability that two neighbors of a given node are themselves neighbors. The shortest path of node *i* was quantified as the averaged number of links traversed along the shortest path between node *i* and all other nodes. Betweenness centrality is the extent to which a given node *i* lies on the shortest paths between node *s* and *t*, and is given by the sum of ratio *n_st_^i^* to *g_st_*. If node *i* lies on the shortest path from node *s* to node *t*, *n_st_^i^* is 1, otherwise 0. *g_st_* is the total number of shortest paths from node *s* to *t*. Eventually, betweenness centrality for node *i* are calculated for and averaged over all possible pairs of nodes other than *i* in the network. Closeness centrality measures the inverse of the sum of path lengths from a given node to other nodes. Degree, clustering coefficient, shortest path length, betweenness centrality and closeness centrality were calculated per node, and were averaged over nodes in each network. We assumed neither directed links nor self-links.

### Statistics

In the conventional analysis, a mixed design two-way ANOVA for number of errors, latency and travel distance in the training phase, and a mixed design two-way ANOVA for time spent around each hole in the probe test were applied. If significant differences were detected in either or both of the interaction and main effects, Tukey’s HSD test was performed as multiple comparisons. In strategy analysis and network analysis, depending on the number of groups, either the Wilcoxon rank-sum test or Kruskal–Wallis test with Bonferroni correction was applied for the usage of each strategy and in network measures between groups each day, respectively. If a significant difference was found in Kruskal–Wallis test, Wilcoxon rank-sum test with Bonferroni correction was applied as a *post hoc* comparison. For all statistical analyses, the significance level before Bonferroni correction was set at 0.05. For ANOVA and Wilcoxon rank-sum test, *η_p_*^2^ and *r* was calculated as effect size, respectively. For strategy and network analysis, statistical results were summarized in Extended Data Figures 3-3, 4-2, 4-3, 5-2, 5-3, 5-4, 5-7, 5-8 and 5-9.

### Code accessibility

All codes for data acquisition and analysis were developed in LabVIEW 2013, 2017 (National Instruments) and MATLAB R2018a (MathWorks Inc.) on a custom-built workstation [Windows 10, Intel(R) Core(TM) i7-7700K CPU @ 4.20 GHz, 32.0 GB RAM]. The code described in the paper is freely available online at https://github.com/suzuki-yusuke/BM_experiment.git, https://github.com/suzuki-yusuke/BM_analysis.git or Extended Data 1.

## Results

### Characteristics of spatial learning in the BM3

First, we explored whether spatial learning in the mice naive for the Barnes maze (BM) is calibrated by scale spaces, comparing the behavioral performances within the BM3 with those in the BM1. The pooled data of Cohort 1 (*n* = 20) and those of the first instance in Cohort 5 (*n* = 14) were assigned to the BM1 group, while the pooled data of Cohort 2 (*n* = 20), Cohort S1 (*n* = 16), and those of the first instance in Cohort 3 (*n* = 20) were assigned to the BM3 group (Table 1). Because Cohort S1 was administered scopolamine hydrobromide at the probe test, the data only in the training phase were used.

### Extensive exploration in the BM3 across the training days

**Figure 3.**
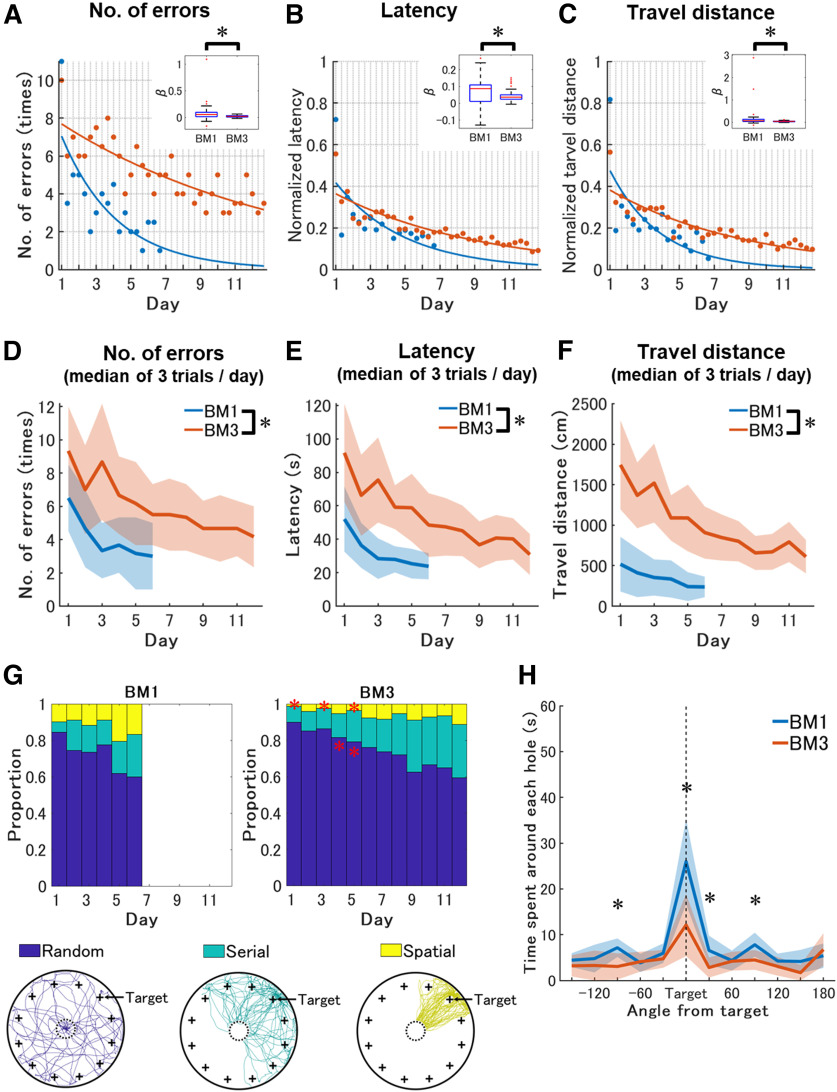
Lower learning rate and inaccurate spatial representation in the BM3 compared with the BM1. ***A–C***, Trial-based learning rate across training periods in conventional features in the BM1 and the BM3. Three trials per day were conducted for six successive days in the BM1 and 11 successive days in the BM3. The measured values of number of errors (***A***), and the normalized values of latency (***B***) and travel distance (***C***) to reach the goals were displayed. The horizontal axis and the vertical gray grid lines of each panel indicates training days and trials, respectively. Note that both latency and travel distance were normalized so that the values range between 0 and 1. Blue-colored and red-colored dots or lines represent the results of BM1 (*n* = 34) and BM3 (*n* = 56), respectively. Each dot represents the median value of each group in each trial. Solid lines represent learning curves estimated by nonlinear exponential curve fitting for the median of the groups. Box plots in the small insets in (***A–C***) represent distributions and comparisons of decay parameter β of the learning curves between the mouse group of BM1 and BM3. Asterisks indicate statistically significant differences. Learning curves fitted for raw values of latency and travel distance are shown in Extended Data Figure 3-2*A*,*B*. ***D–F***, Daily basis learning curves across training periods in conventional features in the BM1 and the BM3. These scores were averaged over three trials per day. Blue and red solid lines represent changes in the median of measured values of number of errors (***A***) latency (***B***) and travel distance (***C***) of the BM1 and the BM3, respectively. Shaded areas indicate median absolute deviation within a mouse group each day. Asterisks indicate significant differences between the BM1 and the BM3. ***G***, Strategy usage of the subjected mice across training days in the BM1 and the BM3. The left and right panel indicate the results of BM1 and the BM3. The vertical and horizontal axes are the proportion of the strategies and training days, respectively. In each stacked bar graph, blue, green, and yellow color represent random, serial and spatial strategy, respectively. Red asterisks indicate significantly different strategy usage between the BM1 and the BM3 in a given day. Samples of each strategy observed in the BM3 are shown under the stacked bar graphs. From left, random, serial spatial strategies are shown. Each colored line represents a trajectory classified as a strategy in a trial. These samples were chosen so that the sum of travel distances of samples within each strategy were comparable between the strategies. All trajectories were transformed so that the goal is located at the right top hole, noted as “Target.” The larger circles with black solid lines represent the edges of the BM3, while the smaller circles with black dashed lines represent the start areas. “+” markers represent hole locations. ***H***, Time spent around each hole in the probe test. The horizontal axis indicates the locations of the holes expressed as angle differences from the target. The vertical and horizontal axes are search time for the individual hole and hole location indicated by angle from the target with 30° step, respectively. “Target” shown by a black dashed line is the goal hole for each mouse. Blue and red represent the results of BM1 (*n* = 34) and BM3 (*n* = 40), respectively. The solid lines are connected between the median values in each angle of each mouse group. The shaded areas are a range of median ± median absolute deviation of the data in each angle. Asterisks indicate significant differences between the BM1 and the BM3. We confirmed that these results were replicated even under durations such that the number of errors were statistically comparable between the BM1 and the BM3 (Extended Data Fig. 3-2*C*). The focal search patterns in scopolamine-treated mice, as reported in the BM1 (Suzuki and Imayoshi, 2017), were partially replicated in the BM3 (Extended Data Fig. 3-4).

To evaluate learning rate across training in the BM1 and the BM3 group, three conventional features (Extended Data Fig. 3-1), the number of errors, latency, and travel distance, were calculated from moving trajectories on the field per training trial, then a learning curve was determined for each mouse (Fig. 3*A–C*). To calculate the learning curves of latency and travel distance between the BM1 and BM3 in unitless form, these were normalized within individuals such that the sum of values across trials in each learning curve becomes 1 (Fig. 3*A–C*). The results of unnormalized latency and travel distance are displayed in Extended Data Figure 3-2*A*,*B*. Then, each learning curve from day 1 to 6 was fitted to an exponential function, *y = αe^-βt^*, estimating optimal intercept *α* and decay parameter *β*, in nonlinear least squares method, where *t* is a trial, *y* is a value of a feature, and *e* is Euler’s number. If *β* was lower, the learning curve takes longer time to converge. Wilcoxon rank-sum test reported that decay parameter *β* in the learning curve of the number of errors, latency and travel distance was significantly lower in the BM3 than in the BM1, *z *=* *2.87, *p *=* *0.00, *r *=* *0.30, *z *=* *2.11, *p *=* *0.03, *r *=* *0.22, *z *=* *3.02, *p *=* *0.00, *r *=* *0.18, respectively (Fig. 3*A–C*). The daily basis learning curve was calculated for each mouse by averaging values across three trials within each day from day 1 to day 6, then compared between the BM1 and the BM3. As expected, the BM3 required a significantly higher number of errors, longer latency and longer travel distance until mice reached the goal, regardless of training days (Fig. 3*D–F*). In a mixed-design two-way [Scale (BM1, BM3) × Day (1–6)] ANOVA for the number of errors, latency and travel distance, the main effect of the scale was significant, *F*_(1,88)_ = 21.11, *p *=* *0.00, *η_p_^2^* = 0.19, *F*_(1,88)_ = 59.82, *p *=* *0.00, *η_p_^2^* = 0.40, *F*_(1,88)_ = 19.08, *p *=* *0.00, *η_p_^2^* = 0.18, respectively. Thus, the learning rate was lower in the BM3 than in the BM1, therefore spatial learning takes a longer time to be established in the BM3 than in the BM1.

10.1523/ENEURO.0505-22.2023.f3-1 Extended Data Figure 3-1. Summary for behavioral features. Download Figure 3-1, DOC file.

10.1523/ENEURO.0505-22.2023.f3-2 Extended Data Figure 3-2 Learning curves fitted for raw values of latency and travel distance and time spent around each hole given adjusted duration. While the BM1 and the BM3 would have different scale lower boundaries of latency and travel distance, respectively, while latency and travel distance should be compared between the BM1 and the BM3 within the same scale. So, latency and travel distance were normalized across trials so that the values range between 0 and 1 (Fig. 3*B,C*). ***A***, A learning curve for raw values of latency. The vertical and horizontal axis indicates latency in second and training days, respectively. The same curve fitting method was used as Figure 3*B*. ***B***, A learning curve for the raw values of travel distance. The vertical and horizontal axis indicates travel distance in centimeters and training days, respectively. The same curve fitting method was used as Figure 3*C*. The small inset in panels ***A*** and ***B*** is the distribution of estimated decay parameter β in the BM1 and the BM3. Asterisk indicates significant differences between the BM1 and the BM3. ***C***, Time spent around each hole in the probe test under adjusted duration for the BM1 and the BM3. Duration for the BM1 and the BM3 was 90 s and 150 s, respectively, and was balanced so that the number of errors were statistically comparable between the BM1 and the BM3. Other than that, the graph format is identical to Figure 3*H*. Download Figure 3-2, TIF file.

10.1523/ENEURO.0505-22.2023.f3-3 Extended Data Figure 3-3 Statistical results of strategy analysis for the BM1 and the BM3 in the training phase. Download Figure 3-3, DOCX file.

**Figure 4.**
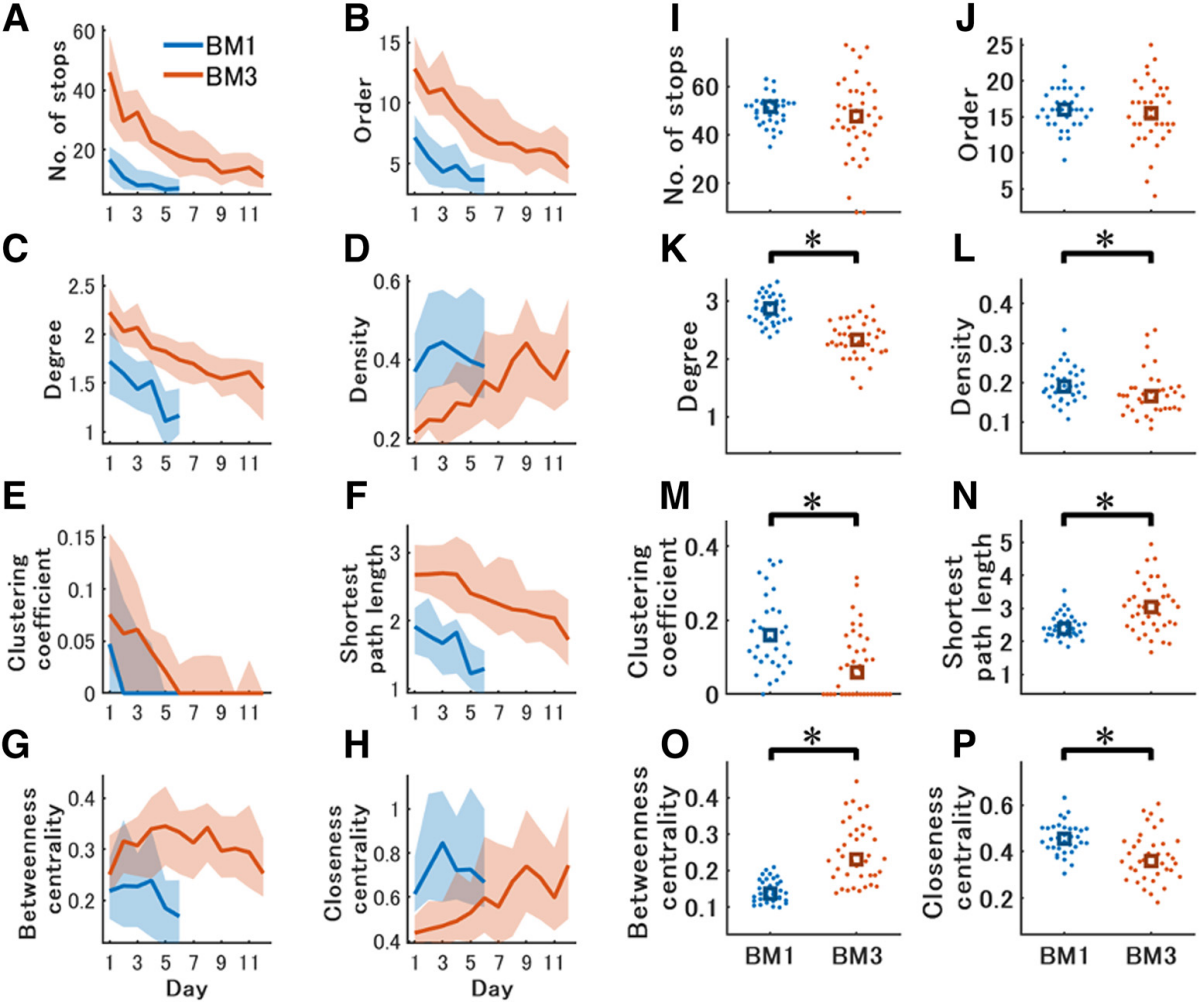
Difference of network structures between the BM1 and the BM3. ***A–H***, Changes of network features across training in the BM1 and the BM3. Temporal changes in the graphically displayed global networks in the BM1 and BM3 during spatial learning were illustrated in Extended Data Figure 4-1. The vertical and horizontal axes in each panel are calculated values of each network feature and training days. These scores were averaged over three trials per day. Blue and red represent the results of BM1 (*n* = 34) and BM3 (*n* = 56), respectively. Solid lines represent changes in the median value of each group in each day. Shaded areas represent error ranges between 25th and 75th percentile of the data within a group each day. Statistical results on each day are shown in Extended Data Figure 4-2. ***I–P***, Network features in the probe test in the BM1 and the BM3. The vertical axis in each panel is each network measure. The blue and red represent the results of the BM1 and BM3 group, respectively. Each dot represents a mouse in either group. Squares represent median value in each group. Asterisks indicate significant differences between the BM1 and the BM3.

10.1523/ENEURO.0505-22.2023.f3-4 Extended Data Figure 3-4 Effects of scopolamine administration on the BM3 probe test. Time spent around each hole in the probe test. Asterisks indicate significant differences between the two groups. Red and blue represent the scopolamine-treated (SCOP) and untreated (NO-SCOP) mouse groups, respectively; the former is cohort S1 (n = 16), while the latter is the pool of Cohort 2 (n = 20) and the first instance of Cohort 3 (n = 20) in Table 1.. A mixed-design 2-way [Scopolamine (SCOP, NO-SCOP) × Hole (1∼12)] ANOVA for the time spent around each hole detected significant interaction between the scopolamine and hole, *F*_(11,594)_ = 2.40, *p *= 0.01, *η_p_*^2^ = 0.04. Multiple comparison testing detected that the time spent around target +30° was significantly longer in the SCOP than in the NO-SCOP whilst time spent around the opposite hole from the target was significantly shorter in the SCOP than in the NO-SCOP. Because a dummy escape tunnel was attached under the hole opposite to the goal hole (see Material and Methods), the NO-SCOP but not SCOP mice might search around it, once they observed that the true escape tunnel no longer exists at the goal hole. Download Figure 3-4, TIF file.

Strategy analysis was performed to qualitatively evaluate how mice optimize their navigation during spatial learning on the BM task (Barnes, 1979; Bach et al., 1995; Eales et al., 2014; Suzuki and Imayoshi, 2017). If the mice have optimized their navigation strategy through the training, they would move along the straight line from the start to the goal (spatial strategy). If mice have incomplete knowledge about the maze and the task (e.g., the goal hole is any one of holes located around the edges of the field), they would sequentially visit each hole toward the goal (serial strategy). If mice were almost naive for the maze and the task, they would show an indeterminate pattern in the trajectory (random strategy). Complete definition of each strategy was described in Extended Data Figure 3-1. The usage of each strategy was compared between the BM1 and the BM3 within each day from day 1 to day 6 (Fig. 3*G*). The usage of spatial strategy on days 1, 3, and 5 was significantly lower in the BM3 than in the BM1 (Extended Data Fig. 3-3, references #13, 15, 17), while that of random strategy on days 5 and 6 was significantly higher in the BM3 than in the BM1 (Extended Data Fig. 3-3, references #5, 6), suggesting that the BM3 would prompt the mice to take more trial-and-error, and would require larger computational resources to optimize navigation strategies than in the BM1.

In the probe test, mixed-design two-way [Scale (BM1, BM3) × Hole (1–12)] ANOVA for the time spent around each hole reported a significant interaction between scale and hole, *F*_(11,792)_ = 11.87, *p *=* *0.00, *η_p_^2^* = 0.14. Multiple comparisons detected that exploration time around the target hole was significantly longer than those around other holes in both BM1 and BM3, while those around 2 holes (target and target +30°) were significantly shorter in the BM3 than in the BM1 (Fig. 3*H*). Also, time around the target ±90° holes were significantly longer in the BM1 than in the BM3. Because a spatial cue was located beyond each target hole, the target ±90° and +180° in the BM1, the BM1 mice might preferentially search around these cues, when they observed that the goal no longer existed. Thus, retrieval of spatial representation was more inaccurate in the BM3 than in the BM1.

We also compared the number of errors and latency between the BM1 and the BM3 in the probe test. The definition of the number of errors was the same as that in the training, while latency in the probe test was defined as the duration from trial start to the moment that the mice initially stayed around the goal hole >10 s in a row, because the escape box and tunnel was removed in the probe test (see Materials and Methods). Latency in the BM3 was approximately three times longer than that in the BM1 on average; median and median absolute deviation of the latency in the BM1 were 28.13 ± 11.78 s while those in the BM3 were 83.13 ± 25.93 s (Student’s *t* test, *t*_(72)_ = −6.45, *p *<* *0.05, *r *=* *0.61). As expected, the latency until mice initially found the goal would depend on the size of scale space. The number of errors during the probe test was significantly lower in the BM3 than in the BM1, median and median absolute deviation of the number of errors in the BM1 were 27 ± 5.5, while those in the BM3 were 16 ± 4 (Student’s *t* test, *t*_(72)_ = 7.08, *p *<* *0.05, *r *=* *0.64). Unlike latency, the number of errors during the probe trial decreased if the scale space increased, because of physical factors such as increased distance to the holes in the BM3, rather than psychological factors such as accurate spatial representation of mice in the BM3.

Comparing the time spent around each hole under normalized durations such that the number of errors are balanced between the BM1 and the BM3 would be also informative, to confirm that the shorter exploration time around the target hole in the BM3 than in the BM1 (Fig. 3*H*) could be because of the poorer spatial representation. We sought the durations of the probe test for the BM1 and the BM3 such that the number of errors were statistically comparable between the BM1 and the BM3, consequently it was 90 and 150 s for the BM1 and the BM3, respectively, then time spent around each hole in each duration were compared. Mixed-design two-way [Scale (BM1, BM3) × Hole (1–12)] ANOVA reported a significant interaction between scale and hole, *F*_(11,792)_ = 4.62, *p *=* *0.00, *η_p_^2^* = 0.06 (Extended Data Fig. 3-2*C*). Even if the same number of errors were allowed in the BM1 and the BM3, we confirmed that the exploration time of the target hole was significantly lower in the BM3 than in the BM1, suggesting the poorer spatial representation in the BM3 than in the BM1.

A previous study reported that mice treated with scopolamine hydrobromide, a nonselective muscarinic acetylcholine receptor antagonist, focally searched holes around the goal and sparsely searched holes far from the goal, compared with vehicle-treated mice in the probe test in the BM1 (Suzuki and Imayoshi, 2017). Such a focal search pattern in scopolamine-treated mice was also observed in the Morris water maze (Huang et al., 2011; Lo et al., 2014). In this study, we examined whether such a search pattern is maintained in the BM3. We found that this search pattern was partially replicated: the scopolamine-treated mice showed significantly longer exploration time in the hole neighboring the goal as well as shorter exploration time in the hole opposite to the goal, compared with nontreated mice (Extended Data Fig. 3-4). These results indicate relatively fewer contributions of cholinergic neurons to the computation in the BM3 than that in the BM1.

### Divergence of network structures between the BM1 and the BM3 along with learning progression

A moving trajectory of a rodent in a field can be expressed as a network with nodes and links, where a node was defined as *xy* coordinates that rodents stayed for certain time, and a link was defined as a transition between two nodes (Weiss et al., 2012; Suzuki and Imayoshi, 2017). The structure of a network was quantified by a set of measures (for complete description of network analysis, see Extended Data Figure 3-1 and Materials and Methods), and shed light on novel aspects of spatial navigation and learning in rodents (Weiss et al., 2012; Suzuki and Imayoshi, 2017) Indeed, a previous study has demonstrated that network structures were simplified across spatial learning in the BM1 (Suzuki and Imayoshi, 2017). Here, we performed this network analysis to explore what kind of structure of networks diverge between the BM3 and the BM1 along with training days 1–6 (Extended Data Fig. 4-1).

In the training phase, the number of stops, order, degree, and shortest path length were significantly and constantly higher in the BM3 than in the BM1 in all training days (Fig. 4*A–C*,*F*; Extended Data Fig. 4-2, references #1–18 and 33–38). These results indicate that the networks generated in the BM3 had more nodes and links, while requiring more traverses between arbitrary two nodes than those in the BM1.

10.1523/ENEURO.0505-22.2023.f4-1 Extended Data Figure 4-1 Visualization of temporal changes in the exploration networks during spatial learning in the BM1 and BM3. ***A***, Temporal changes in the global exploration networks in the BM1 spatial learning. Network structures of exploratory behaviors formed by the dynamic node generation method are plotted (Suzuki & Imayoshi, 2017). Small colored dots and light gray lines represent nodes and links in each mouse (local network). The nodes are located on Cartesian coordinates of the BM1. Nodes are color-coded depending on their polar coordinates. To visualize the global network structure, all local networks of the mice on a single training day were projected on a single plane. Colored larger circles and dark gray lines are global nodes and links in global networks, respectively. Local nodes belong to any one of the global nodes. Likewise, a set of local links are summarized as a global link. The size of a global node is based on log-transformation of the number of nodes that belong to the global node. Likewise, the thickness of a global link is log-transformation of the number of links that belong. ***B***, Topological expression of BM1 global networks. Global nodes were sorted by rank-order of degree and plotted on polar coordinates, so that the node with the highest degree was located at 0 degrees while the lowest one was located at 360 degrees. Circles and lines represent global nodes and links, respectively. Global nodes were ranked to any one of 30 ranks depending on its degree within each group. Then, they were colored according to the rank; the rank first node has a lot of links and is colored by white, and rank 30th node has few links and is colored by black. ***C***, ***D***, Temporal changes in the global networks and their topological expressions of the BM3 spatial learning were displayed. Download Figure 4-1, TIF file.

10.1523/ENEURO.0505-22.2023.f4-2 Extended Data Figure 4-2 Statistical results of network analysis for the BM1 and the BM3 in the training phase. Download Figure 4-2, DOCX file.

10.1523/ENEURO.0505-22.2023.f4-3 Extended Data Figure 4-3 Statistical results of network analysis for the BM1 and the BM3 in the BM1 probe test. Download Figure 4-3, DOCX file.

The density and closeness centrality across training days would initially diverge but eventually converge between the BM1 and the BM3 (Fig. 4*D*,*H*). Both were significantly lower in the BM3 than in the BM1 until day 5 (Extended Data Fig. 4-2, references #19–23 and #47–51), indicating that the observed links in the network in the BM3 accounted for a smaller part of theoretically possible all links, relative to those in the BM1. We confirmed whether the measures changed across days within each group. Density and closeness centrality was comparable between days 1 and 6 in the BM1 (Extended Data Fig. 4-2, references #25, 53), while that was significantly higher on day 6 than on day 1 in the BM3 (Extended Data Fig. 4-2, references #26, 54). Conversely, betweenness centrality diverged along with learning progression (Fig. 4*G*), as it was comparable between the BM1 and the BM3 on day 1, while those were higher in the BM3 than in the BM1 on subsequent days (Extended Data Fig. 4-2, references #39–44). In the BM3, the betweenness centrality represented an arch-like curve across training days, and was comparable between days 1 and 12 (Extended Data Fig. 4-2, reference #46). In contrast, that was significantly lower on day 6 than on day 1 in the BM1 (Extended Data Fig. 4-2, reference #45). Betweenness centrality is a probability such that a node locates on a shortest path between any other two nodes in a network. One interpretation of the results is that certain stopping places relaying any other stopping places were constantly required across training in the BM3, while those could become unnecessary along with training in the BM1. Thus, changes of density, closeness centrality and betweenness centrality across training might depend on both scale space and learning progression.

In the probe test, we found large differences in network structures between the BM1 and the BM3. Degree, density, clustering coefficient, and closeness centrality was significantly lower in the BM3 than in the BM1 (Fig. 4*K–M*,*P*; Extended Data Fig. 4-3, references #3, 4, 5, 8). These results indicated that the nodes were sparsely linked to others in the BM3 than in the BM1. This leads to a significantly longer shortest path in the BM3 than in the BM1 (Fig. 4*N*; Extended Data Fig. 4-3, reference #6). As in the later phase of training, betweenness centrality was significantly higher in the BM3 than in the BM1 (Fig. 4*O*; Extended Data Fig. 4-3, reference #7), suggesting the existence of more stopping places on a shortest path between other 2 stopping places in the BM3 than in the BM1. In contrast, the number of stops and order were comparable between the BM1 and the BM3 (Fig. 4*I*,*J*; Extended Data Fig. 4-3, references #1, 2). One possible explanation is that when mice explored for a given time, the number of stopping places is constant regardless of scale spaces, while the transition patterns between them were modulated by scale spaces.

### Spatial learning between the BM1 and the BM3

Next, we explored whether scale spaces calibrate not only the present but also subsequent spatial learning. When animals learn a task, they learn not only solutions to the immediate task, but also how to find the solutions. This meta-learning process facilitates subsequent learning in variants of the initially learned task. Thus, if this meta-learning process can be activated even for spatial learning between different scale BMs, prior learning in one BM scale (e.g., BM3) would facilitate subsequent spatial learning in another BM scale (e.g., BM1).

### Prior learning in the BM3 facilitated subsequent spatial learning in the BM1

First, we evaluated facilitation of prior spatial learning in the BM3 on the subsequent learning in the BM1. Cohort 3 (*n* = 20) engaged in the BM3 task as the first instance, then the BM1 task as the second instance (Table 1). Cohort 4 (*n* = 17) was a control that engaged in the BM1' task as the first instance then in the BM1 task as the second instance. The BM1' was a variant of the BM1, namely the apparatus other than distal cues was identical to the BM1 (Extended Data Fig. 5-1*A*), and the task lasted for 12 d such that the number of training days matched with the BM3 task. Thus, Cohort 3 and four were treated as the BM3 learners and the BM1' learners, respectively (Fig. 1*A*). The pool of Cohort 1 and the first instance of Cohort 5 was treated as the BM1 beginners (*n* = 34). The performances of BM1 were compared between the BM3 learners, BM1' learners and the beginners. We hypothesized that if prior BM1' or BM3 learning is sufficient for facilitating subsequent spatial learning on the BM1, the BM learners would outperform the BM beginners in the subsequent BM1 task. We confirmed that spatial learning could be established in the BM1' task at the first instance of Cohort 4, similarly to the BM1 task (Extended Data Fig. 5-1*B*).

**Figure 5.**
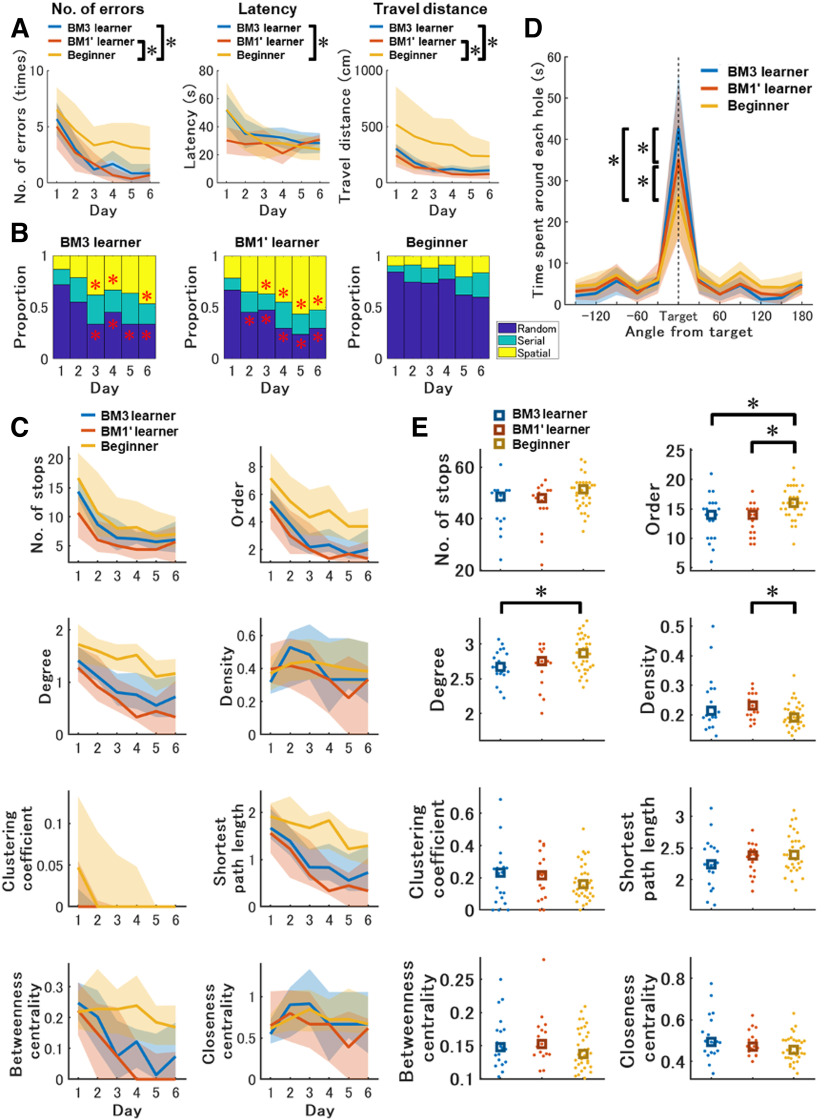
Effects of prior learning in the BM3 and BM1' on spatial navigation and learning in the subsequent BM1 task. ***A***, Daily basis learning curves across training periods in conventional features in the BM1. These scores were averaged over three trials per day. From left, the measured values of number of errors, latency and travel distance are displayed. The mouse groups of BM3 learner (*n* = 16) and BM1' learner (*n* = 17), which experienced the BM3 and BM1' before the BM1, respectively, were compared with the Beginner (*n* = 34) group. The number of errors and travel distances of the BM3 learner and the BM1' learner were significantly lower than Beginner. Latency in the BM1' learner was significantly shorter than beginners. Asterisks indicate statistically significant differences. ***B***, Daily strategy usage during BM1 training in the BM3, BM1' learner and Beginner mouse groups. Although usages of all navigation strategies were comparable between the BM3 and the BM1' learner in all training days, significant differences were detected in the comparison of BM3 learner and Beginner, and BM1' learner and Beginner. Red asterisks indicate significantly different strategy usages compared with the corresponding strategy usages in the beginner on a given day. ***C***, Temporal changes of network features in the BM1 training of BM3, BM1' learner and Beginner. The vertical and horizontal axes in each panel are calculated values of each network feature and training days, respectively. These scores were averaged over three trials per day. Solid lines represent changes in the median value of each group in each day. Shaded areas represent error ranges between 25th and 75th percentile of the data within a group each day. Statistical results on each day are shown in Extended Data Figure 5-3. ***D***, Time spent around each hole in the BM1 probe test of the BM3, BM1' learner and Beginner mouse group. The vertical and horizontal axis is time and hole location indicated by angle from the target with 30° step, respectively. “Target” expressed as a black dashed line is the goal hole for individual mice. Blue, red, and yellow represent the results of Beginner, BM3 learner and the BM1' learner, respectively. The solid lines are median values in each angle in each group. The areas are a range of median ± median absolute deviation of the data in each angle in each group. Asterisks indicate significant differences between the groups. ***E***, Network features in the BM1 probe test compared between Beginner, BM3 learner and the BM1' learner. The vertical axis in each panel is each network measure. The blue, red, and yellow represent the results of Beginner, BM3 learner and the BM1' learner, respectively. Each dot represents a mouse in each group. Squares represent median value in each group. Asterisks indicate significant differences between the mouse groups. We confirmed that the learning curves in the BM1' were virtually similar to those in the BM1 (Extended Data Fig. 5-1). As expected, the facilitation effect from prior learning to the subsequent BM1 learning disappeared if environmental or task structures differed between prior learning and the BM1 learning (Extended Data Fig. 5-5). Not predicted by our model, facilitation effect would be limited, if prior BM learning was done in a smaller scale space than that in the subsequent BM learning (Extended Data Fig. 5-6).

We found that both BM learners exhibited efficient navigation in the training of the BM1, compared with the beginners, as predicted by our model (Fig. 5*A*). In conventional analysis, mixed-design two-way [Instance (BM3 learner, BM1' learner, beginner) × Day (1–6)] ANOVA for number of errors, latency and travel distance detected significant main effect of instance, *F*_(2,68)_ = 9.03, *p *=* *0.00, *η_p_^2^* = 0.21, *F*_(2,68)_ = 3.99, *p *=* *0.02, *η_p_^2^* = 0.11, *F*_(2,68)_ = 8.33, *p *=* *0.00, *η_p_^2^* = 0.20, respectively. Tukey’s HSD test reported that the number of errors and travel distance in the BM learners were significantly lower than the beginners. Latency in the BM1' learners was significantly lower than the beginners.

10.1523/ENEURO.0505-22.2023.f5-1 Extended Data Figure 5-1 The experiment setup and spatial learning curves in BM1 and BM1'. ***A***, Spatial cues used in the BM1 (left panel) and the BM1' (right panel) were indicated with orange arrows. Note that all setups other than the cues were identical between the two mazes. ***B***, Daily basis learning curves across training periods in conventional features in the BM1 and BM1'. These scores were averaged over 3 trials per day. From left, the measured values of number of errors, latency and travel distance are displayed. Only in a mixed-design 2-way [Spatial cues (BM1, BM1') × Day (1–6)] ANOVA for travel distance, the main effect of spatial cues was significant, *F*_(1,49)_ = 6.72, *p *=* *0.01, *η_p_^2^* = 0.12. Asterisks indicate significant differences between the two groups. Download Figure 5-1, TIF file.

10.1523/ENEURO.0505-22.2023.f5-2 Extended Data Figure 5-2 Statistical results of strategy analysis for the BM1’ learner, the BM3 learner, and the Beginner in the BM1 training. Download Figure 5-2, DOCX file.

10.1523/ENEURO.0505-22.2023.f5-3 Extended Data Figure 5-3 Statistical results of network analysis for the BM1’ learner, the BM3 learner, and the Beginner in the BM1 training. Download Figure 5-3, DOCX file.

10.1523/ENEURO.0505-22.2023.f5-4 Extended Data Figure 5-4 Statistical results of network analysis for the BM1’ learner, the BM3 learner, and the Beginner in the BM1 probe test. Download Figure 5-4, DOCX file.

10.1523/ENEURO.0505-22.2023.f5-5 Extended Data Figure 5-5 Prior fear-conditioning experience does not impact on the subsequent BM1 spatial learning. Daily basis learning curves of the CFC learner *n* = 8) and Beginner (*n* = 34) across training periods in conventional features in the BM1. These scores were averaged over 3 trials per day. From left, the measured values of number of errors, latency and travel distance are displayed. Mixed-design 2-way [Instance (CFC learner, Beginner) × Day (1–6)] ANOVA detected neither main effect of Instance nor interaction between instance and day for all features. Download Figure 5-5, TIF file.

10.1523/ENEURO.0505-22.2023.f5-6 Extended Data Figure 5-6 Limited effects of spatial navigation and learning in the subsequent BM3 task by prior learnings in the BM1. ***A***, Daily basis learning curves across training periods in conventional features in the BM3. These scores were averaged over 3 trials per day. From left, the measured values of number of errors, latency and travel distance are displayed. The mouse group of the BM1 learner (*n* = 13), which experienced the BM1 before the BM3, was compared with the Beginner (*n* = 56) group. Although the significant changes were detected in latency and travel distances, no significant difference was detected in the number of errors. ***B***, Strategy usage across training days in the BM3 and comparison between BM1 learner and Beginner. Limited changes were detected only at the Day 4 results. ***C***, Temporal changes of network features in the BM3 training of BM1 leaner and Beginner. Statistical results on each day are shown in Extended Data Figure 5-8. ***D***, Time spent around each hole in the BM3 probe test. Only significant main effect of instance was observed; exploration time in the BM1 learner was significantly longer than that in the Beginner, regardless of hole location. E, Network features in the BM3 probe test compared between BM1 learner and Beginner. Asterisks indicate significant differences between the two groups. Download Figure 5-6, TIF file.

10.1523/ENEURO.0505-22.2023.f5-7 Extended Data Figure 5-7 Statistical results of strategy analysis for the BM1 learner and the Beginner in the BM3 training phase. Download Figure 5-7, DOCX file.

10.1523/ENEURO.0505-22.2023.f5-8 Extended Data Figure 5-8 Statistical results of network analysis for the BM1 learner and the Beginner in the BM3 training phase. Download Figure 5-8, DOCX file.

10.1523/ENEURO.0505-22.2023.f5-9 Extended Data Figure 5-9 Statistical results of network analysis for the BM1 learner and the Beginner in the BM3 probe test. Download Figure 5-9, DOCX file.

Strategy analysis showed that the BM learners more frequently exhibited the spatial strategy than the beginner group, after day 1 (Fig. 5*B*). Data for statistical analyses in Figure 5*B* are reported in Extended Data Figure 5-2. The usage of spatial strategy was significantly different between instances after day 2. *Post hoc* comparison reported that usage of spatial strategy in the BM3 learner was significantly higher than the beginners on days 3, 4, and 6, while those in the BM1' learner were significantly higher than the beginner after day 2. In contrast, the usage of random strategy was significantly different between instances after day 1. The usage of random strategy in the BM3 learner was significantly lower than the beginners after day 2, while those in the BM1' learner were significantly lower than the beginner after day 1. All strategy usages were comparable between the BM3 and the BM1' learner.

The network structures across training days in the BM learners resembled each other while those were distinguishable from the beginner group (Fig. 5*C*). Data for statistical analysis in Figure 5*C* are reported in Extended Data Fig. 5-3. The number of stops was significantly different between instances on day 4. *Post hoc* comparison reported that the number of stops in the BM1' learner was significantly lower than the beginners. Order and degree in the BM learners were significantly lower than that in the beginner after day 1, suggesting that the BM learners could move between two nodes on the network with fewer traverses of other nodes compared with the beginners. Betweenness centrality in the BM learners was significantly lower than the beginners after day 2, suggesting that the beginners require certain places relaying between other two places on the field compared with the BM learners (Fig. 5*C*). Thus, conventional, strategy, and network analysis suggest that the BM learners could learn the BM1 task efficiently compared with the beginners.

Not predicted by our hypothesis, the BM3 learners exhibited the most accurate spatial memory in the probe test (Fig. 5*D*). Indeed, mixed-design two-way [Instance (beginner, BM3 learner, BM1' learner) × Hole (1–12)] ANOVA for the time spent around each hole, significant interaction between instance and hole was detected, *F*_(22,748)_ = 4.16, *p *=* *0.00, *η_p_^2^* = 0.11. Multiple comparisons detected that the time spent around the target hole in the BM3 learners was significantly longer than all other groups, and the BM1' learner was significantly longer than only the beginners. Thus, prior learning in the BM3 rather than in the BM1' improves spatial representation in subsequent learning in the BM1, suggesting that prior learning in a larger scale space would facilitate subsequent spatial learning in a smaller scale space.

The network analysis revealed significant differences of exploratory networks between instances (Fig. 5*E*). Order, degree, and density were significantly different between instances (Extended Data Fig. 5-4, references #2, 6, 10). Order in the BM learners was significantly lower than the beginner (Extended Data Fig. 5-4, references #4, 5). Degree in the BM3 learner was significantly lower than the beginner (Extended Data Fig. 5-4, reference #9). Density in the BM1' learner was significantly higher than the beginner (Extended Data Fig. 5-4, reference #12). Thus, the BM3 learners would traverse between a limited number of places. These results support the idea that the prior spatial learning in the BM3 most facilitated subsequent spatial learning in the BM1.

Although we explored meta-learning effects only within the BM paradigm, we also checked whether prior learning in another behavioral paradigm such that neither environmental nor task structures were shared with the BM paradigm facilitates subsequent spatial learning in the BM1 task. Cohort S2 (*n* = 8) underwent a conventional contextual fear conditioning (CFC) task at the first instance (see Materials and Methods), then BM1 at the second instance. The CFC learners formed contextual fear memory correctly, as Wilcoxon signed-rank test detected significantly higher freezing response in the fear acquisition context (M = 38.14, SD = 15.27), compared with that in the different context (M = 14.30, SD = 10.21), *p *=* *0.02, *r* = −0.84, *z* = −2.38. The performance in the BM1 was comparable between the CFC learners and the BM1 beginners, as there were no significant differences between the two in all conventional features (Extended Data Fig. 5-5). We confirmed that facilitation by meta-learning occurs if prior and subsequent learning share a common task or environmental structure.

### Limited facilitation from the BM1 to the BM3 learning

To test whether prior learning in the BM1 facilitates subsequent learning in the BM3, the performances in the BM3 at the second instance of Cohort 5 (BM1 learner, *n* = 13) were compared with those in the BM3 beginner (Fig. 1*B*). The beginners in the training were pool of Cohort 2 (*n* = 20), and the first instance of Cohort 3 (*n* = 20) and S1 (*n* = 16), while those in the probe test were pool of Cohort 2 and the first instance of Cohort 3, because Cohort S1 was treated with scopolamine hydrobromide at the probe test.

In conventional analysis, the number of errors were comparable between the BM1 learners and the beginners, while latency and travel distance was significantly shorter in the BM1 than in the beginner, regardless of training days (Extended Data Fig. 5-6*A*). Mixed-design two-way [Instance (BM1 learners, beginners) × Day (1–6)] ANOVA for latency and travel distance detected significant main effect of instance, *F*_(1,67)_ = 28.72, *p *=* *0.00, *η_p_^2^* = 0.30, *F*_(1,67)_ = 24.85, *p *=* *0.00, *η_p_^2^* = 0.27, respectively. These results suggest that the BM1 learners explored places other than the holes less across training days, compared with the beginner, while acquisition of spatial memory in the BM3 would be comparable to that in the beginner.

In the strategy analysis, strategy usage was almost the same between the BM1 learners and the beginners (Extended Data Fig. 5-6*B*). The usage of random strategy was significantly higher in the BM1 learners than in the beginners only on day 4 (Extended Data Fig. 5-7, reference #4).

In network analysis, the number of stops was significantly lower in the BM1 learners than in the beginners on days 1, 3, 4, 7, 8, 11 (Extended Data Fig. 5-6*C*; Extended Data Fig. 5-8, references #1, 3, 4, 7, 8, 11). Although the number of stops were constantly different, order was significantly lower in the BM1 learners than in the beginners only on days 3 and 4 (Extended Data Fig. 5-8, references #15, 16). Degree was significantly lower in the BM1 learners than in the beginners on days 3 and 4 (Extended Data Fig. 5-8, references #27, 28). Clustering coefficient was significantly lower in the BM1 learners than in the beginners only on day 3 (Extended Data Fig. 5-8, reference #51). Shortest path length was significantly lower in the BM1 learners than in the beginners on day 4 (Extended Data Fig. 5-8, reference #64). These suggest that the beginners frequently search within certain places, compared with the BM1 learners. Other than that, strategy and network structures of spatial navigation would resemble between the BM1 learners and beginners (Extended Data Fig. 5-6*B*,*C*).

In the probe test, the BM1 learners explored holes for a longer time than the beginners, regardless of the hole locations (Extended Data Fig. 5-6*D*). In a mixed-design two-way [Instance (beginners, BM1 learners) × Hole (1–12)] ANOVA for the time spent around each hole, the main effect of instance was significant, *F*_(1,51)_ = 7.23, *p *=* *0.01, *η_p_^2^* = 0.12. Multiple comparisons detected that the time spent around each hole was significantly longer in the BM1 learner than in the beginner regardless of the hole location.

Network analysis reported that the number of stops and the order was comparable between the BM1 learners and the BM3 beginners (Extended Data Fig. 5-6*E*; Extended Fig. 5-9, references #1, 2). In contrast, degree, density, clustering coefficient, shortest path length, and closeness centrality was higher in the BM1 learner, while shortest path length was lower in the BM1 learner compared with the beginner (Extended Data Fig. 5-9, references #3, 4, 5, 6, 8). These results suggest that the BM1 learners exhibited more various patterns of transitions between nodes in the networks (Extended Data Fig. 5-6*E*), leading to a longer hole exploration time in the BM1 learners than in the beginners (Extended Data Fig. 5-3*D*). Betweenness centrality was significantly lower in the BM1 learners than in the beginners (Extended Data Fig. 5-9, reference #7), suggesting that the beginners required certain places relaying between two other places (Extended Data Fig. 5-6*E*).

Through the meta-learning experiments between the BM1 and the BM3, we found that the facilitation from prior BM learning to subsequent BM learning is calibrated by scale space. Namely, the prior learning in the BM3 even more than in the BM1' facilitated the subsequent spatial learning in the BM1, meanwhile the facilitation from the prior BM1 learning to the subsequent BM3 learning was limited. A possible interpretation of these results is that prior BM learning facilitates subsequent BM learning performed on equal or smaller scale spaces.

## Discussion

### Spatial learning diverged between the BM1 and the BM3 along with learning progression

Environmental scale space is thought to differentiate the spatial navigation and learning in animals (for review, see Geva-Sagiv et al., 2015). In this study, we developed a larger scale space, 3-m diameter Barnes maze (BM3; Fig. 2*B*,*D*,*F*), and compared the behavioral features in the BM3 with those in a conventional scale space, 1-m diameter Barnes maze (BM1; Fig. 2*A*,*C*,*E*), to explore how scale space calibrates spatial learning within and between the BM tasks. A total of 115 mice were subjected to this study, and each was assigned to any one of seven cohorts (Table 1).

We first demonstrated that spatial learning in the BM3 was successfully established. The learning curves in the conventional features of the number of errors, latency and travel distance were decreased and converged across training (Fig. 3*A–C*). The usage of optimal and suboptimal navigation strategy (i.e., spatial and serial strategy, respectively) increased, while use of naive strategy (i.e., random strategy) decreased across training (Fig. 3*G*). In the probe test, the exploration time in the goal hole was significantly longer than those in the other holes (Fig. 3*D*).

Then, behavioral features were compared between the BM3 and the BM1. In the training phase, all of the number of errors, latency, and travel distance were constantly higher in the BM3 than in the BM1 (Fig. 3*D–F*). The learning curves of them were more gradual in the BM3 than in the BM1 (Fig. 3*A–C*), indicating that the spatial learning in the BM3 takes longer time until convergence than that in the BM1. The usage of spatial strategy was lower, while that of the random strategy was higher in the BM3 than in the BM1 (Fig. 3*G*). The BM task can be viewed as a problem to find optimal navigation strategy (i.e., spatial strategy) through trial-and-error using a variety of strategies. In line with this, the BM3 would demand more trial-and-error iterations to solve this problem. In the probe test, exploration time in the target hole was significantly shorter in the BM3 than in the BM1, while those in the other holes were virtually comparable between the BMs (Fig. 3*H*), even if the number of visiting holes other than the target was balanced between the BM1 and the BM3 (Extended Data Fig. 3-2*C*). Thus, as expected, spatial representation would be more inaccurate in the BM3 than in the BM1, since the BM3 would require more computational resources to establish learning than the BM1. However, to our knowledge, this is the first result demonstrating the effect of space scale on a goal-directed spatial learning, Barnes maze, in mice.

The increase in demand for computational resources in the BM3 could be partially explained by reducing spatial resolution of place cells in larger scale spaces. Several previous studies have reported that the size of place field per place cell in larger spaces was larger than those in smaller spaces (O’Keefe and Burgess, 1996; Fenton et al., 2008; Kjelstrup et al., 2008; Park et al., 2011; Rich et al., 2014; Harland et al., 2021). Nevertheless, in the nature of spatial navigation in animals, multiscale space representation should coexist so that animals can navigate from the order of centimeters to the order of kilometers (Geva-Sagiv et al., 2015). Indeed, a human fMRI study demonstrated a network of brain regions that were activated in either or both the navigation in large and small scale spaces (Li et al., 2021). Hence, we expect that more computational resources might be allocated in the BM3 to process multiscale spatial representations in parallel, while the contribution of cholinergic neurons at least would be relatively fewer (Extended Data Fig. 3-4). The coupling of the BM1 and the BM3 could be exploited for examining neural representations of large and small scale space.

The differences in learning behaviors between the BM1 and the BM3 emerged in the network structures in moving trajectories. In the training, the number of stops, order, degree, and shortest path length was higher in the BM3 than in the BM1 regardless of training days (Fig. 4*A–C*,*F*), suggesting that the BM3 prompted the mice to generate more nodes (i.e., stopping points) on the field and transition between them regardless of learning progression. Density and closeness centrality converged between the BM1 and the BM3, as both increased in the BM3 but were constant in the BM1 across learning progression (Fig. 4*D*,*H*). On the other hand, betweenness centrality diverged between the BMs, as it was constant in the BM3 but decreased in the BM1 (Fig. 4*G*), suggesting that certain stopping places that relay other stopping places in the BM3 persisted, while those in the BM1 would disappear along with learning progression. Thus, density, closeness centrality and betweenness centrality would be representative measures, contrasting spatial learning in larger scale spaces with that in smaller scale spaces.

Particularly, such characteristics of betweenness centrality in the BM3 would signal persistently existing “home bases” in the exploration in the BM3. Ethological studies found that when animals face a visible but unfamiliar environment, they initially acquire spatial representation of the environment through home base behavior, a set of roundtrips arriving and departing on a specific place (i.e., home base) neighboring a starting point, salient landmarks or along the perimeter of the field (Yaski et al., 2011; Eilam, 2014). The route-following is one of strategies establishing the home base behavior, such that animals turn back at the end of the outbound path from the home base, then simply retrace it on the inbound path to the home base, where usually the outbound path is slow with a number of stops, while the inbound path is fast with fewer stops (Eilam, 2014). The BM3 contains salient landmarks, especially the displays surrounding the field (Fig. 2*C*), and the clusters of nodes more than the number of holes existed along with the perimeter of the field (Extended Data Fig. 4-1*C*; cf. Extended Data Fig. 4-1*A*), suggesting that some of these nodes might act as home bases. If mice used route-following strategy, further nodes and links could be generated on an outbound path from the home base nodes, resulting in larger order networks as shown in the early phase of the learning (Fig. 4; Extended Data Fig. 4-1*C*). In the later phase of the learning, the total number of nodes per network was decreased and the network structures converged to a simpler one (Fig. 4; Extended Data Fig. 4-1*C*), as the mice switched the route-following navigation to the map-based navigation. Counterintuitively, certain nodes in the BM3 remained even after the mice acquired the map-based navigation, so as to maintain a constant value of betweenness centrality per network across learning (Fig. 4*G*; Extended Data Fig. 4-1*C*). In contrast, some of such nodes disappeared along with learning progression in the BM1, with the consequence of decreasing betweenness centrality (Fig. 4*G*). To our knowledge, only one ethological study demonstrated that the home base behavior in voles diverged between relatively larger and a smaller scale space within 15 min after the initial exposure to the fields (Eilam et al., 2003). We expect that the presented experimental and analytical suite of the BM3 would be applicable for ethological studies to examine the home base behaviors in longer time scales for days or weeks in the context of spatial learning and memory.

Nodes and links in a navigational network were calculated from stopping coordinates and transitions between nodes, respectively (see Materials and Methods). It can be expected that the brain would represent not only geometric structures (e.g., coordinates, distances, boundaries) of the nodes, but also topological structures (e.g., degree, betweenness, closeness centrality) of them. Early psychological study suggested that rats represent topological structures of a maze of a conventional scale (Poucet and Herrmann, 2001). Recently proposed algebraic topological model predicted that the hippocampal place cell ensembles with a certain firing rate, place field size and population size are capable of encoding topological signatures of environments of conventional scales (2 × 2 m) within several minutes (Dabaghian et al., 2012). Furthermore, one electrophysiological study in rats demonstrated that topological structures of a shape-changeable track (4 m in length) might be encoded in place cells in the hippocampal CA1, as these place fields were preserved even if configuration of the track was varied in Cartesian coordinates, as long as the topological structure was maintained (Dabaghian et al., 2014). For neural correlates in topological expression of larger scale space, one human fMRI study demonstrated that degree and closeness centrality at each street in London’s Soho correlated with the right posterior and right anterior hippocampal activities while participants virtually visited there (Javadi et al., 2017). These data suggest that topological structures or measures are expressed in the hippocampus and presumably CA1 place cells. Topological expression would reduce computational cost for spatial representation, rather than expressing them in a geometrically precise manner (Dabaghian et al., 2014). This benefit would enable a hierarchical network representation of large environments: a large environment is represented by a network such that each node is a spatial representation of a local region in the large environment and each link represents a connection between a pair of the nodes (Poucet, 1993; Wolbers and Wiener, 2014). Thus, the topological coding might be more invoked during navigations in larger scale spaces than in smaller scale spaces, hence the BM3 would be suitable to probe neural representation of topological coding.

### BM learning in a scale space is more facilitated by prior BM learning in equal or larger scale spaces

Next, we explored whether scale spaces calibrate not only the present but also subsequent spatial learning. When animals learn a task, they learn not only immediate task solutions per se, but also how to find the solutions: Harlow’s “learning set” (Harlow, 1949). This meta-learning process leads to subsequent few-shot learning in variants of the initially learned task (for review, see Wang, 2021). Therefore, we hypothesized that if this meta-learning process can be activated over spatial learning in variants of BM tasks, prior learning in one BM task would facilitate subsequent spatial learning in another BM task.

The BM3 learners and the BM1' learners were engaged in the BM3 task and the BM1' task as prior learning, respectively, then both were tested in the BM1 task. The beginners were engaged only in the BM1. As expected, prior learning in the BM tasks facilitates subsequent spatial learning in the BM1. During the training, the BM learners exhibited optimal navigation in the subsequent BM1 task compared with the beginners (Fig. 5*A*,*B*). Not predicted by our hypothesis, the accuracy of spatial representation in the subsequent BM1 was highest in the BM3 learners, as the exploration time around the goal hole was highest in the BM3 learners even more than the BM1' learners (Fig. 5*D*). Thus, prior learning in a large scale space would elaborate spatial representation in the subsequent spatial learning in a smaller scale space.

The facilitation effect between BM1 and BM3 was asymmetric, as the facilitation effects from the prior learning in the BM1 to subsequent learning in the BM3 was limited. The number of errors was comparable between the BM1 learner and the beginner (Extended Data Fig. 5-6*A*), and the usage of the navigation strategies was virtually comparable between the BM1 learners and beginners (Extended Data Fig. 5-6*B*). Meanwhile, the latencies and travel distances were significantly lower for the BM1 learners (Extended Data Fig. 5-6*A*). In the probe test, exploration time of holes was significantly longer in the BM1 learners than the beginners, regardless of hole locations (Extended Data Fig. 5-6*D*). These results indicated that the BM1 learners took less time to explore places other than places around holes in the subsequent BM3 task, since they could exploit incomplete knowledge of the BM task (i.e., the goal always located at any one of peripheral holes) that was acquired through the prior learning in the BM1.

The facilitation of spatial learning between the BM1 and the BM3 also resulted in the differences of network structure during the training, especially betweenness centrality would be a representative measure for a facilitation of spatial learning. In the BM3 to BM1 facilitation, decrease of betweenness centrality in the BM3 learners across training was significant and steeper than that in the beginners (Fig. 5*C*). In the BM1 to BM3 facilitation, such differences compared with the beginners were not observed in the BM1 learners (Extended Data Fig. 5-6*C*). These results suggest that the facilitation from the BM3 to the BM1 would allow the learners to acquire spatial representation of the BM1 while minimizing to generate certain places like home base (see above). In contrast, the facilitation from the BM1 to the BM3 would prompt the learners to generate such places at the same extent with that in the beginner.

Many studies have reported meta-learning in a wide variety of tasks such as schema learning (Tse et al., 2007), multiple decision-making (Rosenberg et al., 2021), structure learning (Braun et al., 2010), and spatial learning (Ocampo et al., 2018; Baraduc et al., 2019; Alonso et al., 2021). In common with these studies, the facilitation from prior learning to subsequent learning was observed if both prior and subsequent learning were performed under a variant of common task rule in virtually the same size of scale space. Our results support the idea that the BM3 and the BM1 are processed as a variant of the same BM task, as shown in the facilitation in the BM learners but not in the CFC learners who engaged in the prior task that shared neither the task structure nor the environmental structure with the BM task. In addition, we found that this facilitation is calibrated by scale spaces at each learning instance, namely, if a scale space of a subsequent learning (i.e., BM1) is equal or smaller than that at a prior learning (i.e., BM3 or BM1'), the facilitation effect becomes obvious (i.e., BM3 or BM1' learners vs beginners in the BM1). In this case, the BM3, BM1', and BM1 task would be processed as a variant of a BM task for the BM learners. In contrast, if a scale space of a subsequent learning (i.e., BM3) is larger than that at a prior learning (i.e., BM1), the facilitation effect is limited (i.e., BM1 learners vs beginners in the BM3).

One possible neural circuit underlying the scale space dependent facilitation in spatial learning would involve the hippocampus and medial prefrontal cortex (mPFC). The hippocampus is activated to encode a novel context, rather than contexts that fit into a previously learned one (for review, see Alonso et al., 2020). Specifically, NMDA receptors in the hippocampal CA3 contribute to novelty detection on the CA1 place cells (Dragoi and Tonegawa, 2013). In a prior and novel BM learning, a memory engram encoding not only spatial representation of the immediate BM, but also a learning set of the BM task would be generated at the hippocampus. Then, the memory engram is transferred from the hippocampus to networks in the cortex through the consolidation process within a couple of weeks. The mPFC would involve classifying whether a current context fits into existing ones, and suppressing hippocampal activity during memory encoding, unless the context is novel and unlike previously acquired one (van Kesteren et al., 2012). If a subsequent BM learning is a variant of the prior BM learning, a completely new memory engram would be generated little at the hippocampus, because the mPFC infers the current context as similar to the prior one, and suppresses the hippocampal activities. The existing memory engram that has been transferred to the cortex is directly activated bypassing the hippocampus, and slightly tuned to solve the task, leading to few-shot learning (Alonso et al., 2020).

Since the BM3 required more trial-and-error and computational resources to learn the task than the BM1 (Fig. 3), a relatively larger memory engram should be recruited for the BM3 than for the BM1, for encoding spatial representation of the maze with a learning set of the task. If mice experienced the BM3 task as a prior learning followed by the BM1 task as a subsequent learning, a larger memory engram is generated at the hippocampus during the prior learning. Then, the memory engram is transferred from the hippocampus to the cortex, through the blank between the prior and subsequent learning. In the subsequent BM1 learning, a part of the already acquired memory engram is slightly tuned to solve the task, while information that had to be encoded as a completely new memory engram in the hippocampus became rare. Owing to this, the BM3 learners could learn the subsequent BM1 task quickly, while the beginners had to generate a new memory engram to the hippocampus. In contrast, if mice experienced the BM1 first, a smaller memory engram is generated, hence only tuning the existing engram would be not enough to learn the subsequent BM3 task where a certain amount of new memory engram in the hippocampus is required, resulting in the limited facilitation by prior BM1 in the subsequent BM3 learning. Thus, the BM3 to BM1 facilitation could induce certain neuronal processing more optimized to solve the task, and thereby be helpful for measuring neural activities essential for spatial navigation.

In conclusion, spatial learning diverged between the BM1 and the BM3, namely the mice in the BM3 persistently explored certain places even in the later phase of learning, so that betweenness centrality in the networks of exploration path were constant throughout the learning. Such prior experience in the BM3 would facilitate subsequent spatial learning in the BM1 suggesting that the BM3 learners acquire more optimized neural computation to solve the task than the beginners. The couple of the BM1 and the BM3 would be a suitable system to examine how animals represent different scale spaces, as well as the underlying neural implementation.

## Synthesis

Reviewing Editor: Christine Portfors, Washington State University Decisions are customarily a result of the Reviewing Editor and the peer reviewers coming together and discussing their recommendations until a consensus is reached. When revisions are invited, a fact-based synthesis statement explaining their decision and outlining what is needed to prepare a revision will be listed below. The following reviewer(s) agreed to reveal their identity: Masaki Kakeyama.

The reviewers agree that the study is potentially interesting but has some issues that need to be resolved. One issue is that the methods and results of the probe trial should be described in more detail. The area of the target field and its difference between the two mazes, and how to normalize the difference should be addressed. In addition, the latency and number of errors at the time of the probe trial should be addressed. Another point relates to providing a discussion on the possibility that the mice in BM3 start by first using a “route following” strategy and then acquire map-based navigation. Overall, substantial revising of the text is necessary to improve the significance of the study and the broader impact of the results. In its current form, the manuscript does not provide a strong rationale for some aspects of the study, is very challenging to read and does not clearly articulate how the results move the field forward.

Specific comments:

The significance statement is not very strong. Could the authors write more on the significance/importance of the results and less on the description of the study?

There is no rationale for cohort 3; mice administered scopolamine hydrobromide

The results that BM3 is more challenging than BM1 are not surprising. It’s a bigger and more challenging space. The significance of this result in a broader context should be provided.

The rationale for the BM1’ trials is not clear.

The description of table 4 in the text is very challenging to read. Perhaps instead of writing out all the results in the text, the authors could just refer to the table for all the individual statistical results. Same thing for table 5.

It seems that many of the results are similar to previous studies as there are many references in the Results section to support the results of the current study. First, this makes those paragraphs challenging to read, 2) it makes the reviewer question what is new in the current study.

The rationale for the contextual fear conditioning test is not clear.

The manuscript in general is challenging to read. There is too much detail in some areas and not enough in others. The data are interesting but need to be described in a broader context to understand their implication.

Regarding probe trial, the reviewer wants to know why the authors take 5 min for probe trial? Based on the BM original repot and the current results in Fig. 2, the reviewer speculate duration of probe trial would better to be around 60 sec and 180 sec, for BM1 and BM3, respectively?

Fig. 5 seem better to be an Extended figure.

The authors seem better to discuss about “route following” strategy of mice. There may be a possibility that Results of BM3 in Extended Figure 3 reflect it. In my point of view, the mice in BM3 seems to use “route following” strategy at first (by using external monitor cues), and then, acquire map-based navigation.

## Author Response

Response to the editor and the reviewers

We thank the editor and the reviewers for their critical and insightful comments.

We answered all comments one by one as below.

The corrections we made are highlighted in the manuscript file by proofing function of Word, and we provide a clean copy of the manuscript file also. Synthesis Statement for Author (Required):

Comment #1

One issue is that the methods and results of the probe trial should be described in more detail.

Reply

The procedure of the probe test was identical to that of the habituation except that the mice were moved to the home cage without staying in the escape box. We have specified this point at line 168. In relation to comment #3 and #13, we additionally calculated the number of errors and latency in the probe test, then time spent around each hole under durations such that the number of errors were statistically comparable between the BM1 and the BM3. These results were described at line 401-427. In relation to comment #13, we specified the length of data in the probe test actually analyzed at line 272.

Comment #2

The area of the target field and its difference between the two mazes, and how to normalize the difference should be addressed.

Reply

We further specified the definition of the target fields in Extended Data Table 1, and inserted it at line 265 and line 1020.

Comment #3

The latency and number of errors at the time of the probe trial should be addressed.

Reply

2

We calculated both the latency and the number of errors during the probe trial, and the results and discussion were inserted to line 401.

In relation to comment #12, we sought the duration for the BM1 and the BM3 such that the number of errors were statistically comparable between the BM1 and the BM3, then compared time spent around each hole in each duration between the BMs (Extended Data Fig 3-1C). We expect that this procedure roughly balanced the extent of hole visits between the BM1 and the BM3 (for detail, see the reply to comment #12).

Comment #4

Another point relates to providing a discussion on the possibility that the mice in BM3 start by first using a “route following” strategy and then acquire map-based navigation.

Reply

Together with comment #15, we appreciate the reviewer for suggesting this insightful comment. Our speculation was provided at line 696 and line 780 in the

Discussion part.

Specific comments:

Comment #5

The significance statement is not very strong. Could the authors write more on the significance/importance of the results and less on the description of the study?

Reply

The three major findings of our study can be summarized as follows. First, the BM learning was established even in 3 m scale space, the largest of its kind in the world.

Second, we found that spatial learning diverged between the BM1 and the BM3, in other words the mice in the BM3 explored particular places even in the later phase of learning, so that betweenness centrality in the networks of exploration path were constant along with learning progression. Third, such prior experience in the BM3 facilitates subsequent spatial learning in the BM1, suggesting more optimized neural computation than the beginners. We demonstrated that the coupling of the BM1 and the BM3 would be a suitable system that is applicable for ethological or neuroscientific studies examining how animals represent different scale spaces with the underlying neural implementation. This point is the significant statement of this study. We described these statements throughout the manuscript, especially in the Abstract,

3

Results and Discussion, while we removed redundant descriptions in the manuscript, especially in the Material and Methods, the Results, and the Discussion.

Comment #6

There is no rationale for cohort 3; mice administered scopolamine hydrobromide.

Reply

We inserted the purpose, results and discussion for Cohort 3 at line 109, line 680, line 1104, while simplifying the descriptions. Also, we changed Cohort 3 to Cohort S1, and thereby the numbering of other cohorts, too (Cohort section in Material &

Methods part and Table 1).

Comment #7

The results that BM3 is more challenging than BM1 are not surprising. It’s a bigger and more challenging space. The significance of this result in a broader context should be provided.

Reply

The significance of the results of the BM3 in the context of ethology (e.g. line 696) and neuroscience (e.g. line 837) was described more explicitly in the Discussion part.

Comment #8

The rationale for the BM1’ trials is not clear.

Reply

We inserted the purpose, results and discussion for Cohort 4 (in the latest number) at line 106 and line 514.

Comment #9

The description of table 4 in the text is very challenging to read. Perhaps instead of writing out all the results in the text, the authors could just refer to the table for all the individual statistical results. Same thing for table 5.

4

Reply

We removed the description of each statistical test and results in Statistical table 4 and 5 from the manuscript, then inserted the descriptions “Data for statistical analyses in Fig. 5B are reported in Extended Data Table 5.” to line 528, as well as “Data for statistical analysis in Fig. 5C are reported in Extended Data Table 6.” to line 538, as has been done in previous eNeuro articles (Tyssowski and Gray, 2019; Backer et al., 2020). Tyssowski KM, Gray JM. Blue Light Increases Neuronal Activity-Regulated Gene Expression in the Absence of Optogenetic Proteins. eNeuro. 2019 Sep 18;6(5):ENEURO.0085-19.2019. doi: 10.1523/ENEURO.0085-19.2019. PMID: 31444226; PMCID: PMC6751372.

Backer, K. C., Buchsbaum, B. R., & Alain, C. (2020). Orienting Attention to Short-Term Memory Representations via Sensory Modality and Semantic Category Retro-Cues. eNeuro, 7(6), ENEURO.0018-20.2020. https://doi-org.kyoto-u.idm.oclc.org/10.1523/ENEURO.0018-20.2020

Comment #10

It seems that many of the results are similar to previous studies as there are many references in the Results section to support the results of the current study. First, this makes those paragraphs challenging to read, 2) it makes the reviewer question what is new in the current study.

Reply

We have tried to emphasize the significance of this study throughout the manuscript, especially in the Abstract, Results and Discussion part, whilst comparing our own new findings to the evidence presented in previous studies. Please also refer to our reply to Comment #5

Comments #11

The rationale for the contextual fear conditioning test is not clear.

Reply

We inserted the purpose, results and discussion for Cohort S2 (in the latest number) at line 117 and line 797.

5

Comments #12

The manuscript in general is challenging to read. There is too much detail in some areas and not enough in others. The data are interesting but need to be described in a broader context to understand their implication.

Reply

Please refer to our reply to Comment #5.

Comment #13

Regarding probe trial, the reviewer wants to know why the authors take 5 min for probe trial? Based on the BM original report and the current results in Fig. 2, the reviewer speculate duration of probe trial would better to be around 60 sec and 180 sec, for BM1 and BM3, respectively?

Reply

We appreciate the reviewer for suggesting this critical comment. In this study, we evaluated meta-learning effects between the BM3 and the BM1, hence we controlled the experimental procedures including the duration of the probe test to be as same as possible between the BM1 and the BM3.

Determining durations of the probe test, whether the duration for the BM3 should be simply tripled to that for the BM1 is unobvious. Optimal duration should be determined by at least the dimensions of mice’s cognitive flexibility which is a preference for exploring holes other than the target hole given an observation that the escape box or tunnel no longer exists under the target hole (e.g. Eales et al., 2014), though it is not guaranteed that the cognitive flexibility in each animal is constant across the BM1 and the BM3 learning even in the context of meta-learning.

Therefore, we set a relatively longer 300 s duration for the probe trial to capture behavioral outcomes including cognitive flexibility, while we analyzed the data for the first half (0∼150 s) of each probe trial (300 s in total) for both the BM1 and the BM3, based on our pilot studies. This duration was enough to evaluate the establishment of spatial learning before cognitive flexibility dominates. The duration for analysis was inserted at line 272.

However, we agree that comparing the time spent around each hole, a measure of accuracy of spatial memory in the probe trials, under normalized durations such that the number of hole visits are balanced between the BM1 and the BM3 would be informative, to confirm our results that the shorter exploration time around the target 6 hole in the BM3 than in the BM1 (see p.17 line 394 in the Results part) could be due to psychological factors such as poorer spatial memory in the BM3 learners, rather than physical factors such as increased distance to holes in the BM3. Based on the analysis for comment #2, we sought the duration of the probe test for each of the BM1 and the BM3 such that the number of errors were statistically comparable between the BM1 and the BM3, consequently it was 90 s and 150 s for the BM1 and the BM3, respectively, then time spent around each hole in each duration were compared. We expect that this procedure would roughly balance the number of hole visits between the BM1 and the BM3. As a result, even if the same number of errors were allowed in the BM1 and the BM3, the exploration time of the target hole was significantly lower in the BM3 than in the BM1, as Fig. 2H, suggesting the poorer spatial representation in the BM3 than in the BM1. These results are in Extended Data Fig. 3-1C, and we have described them at line 416.

Though we performed the network analysis for the BM1 and the BM3 in the durations above, all the differences of the network features between the BM1 and the BM3 replicated those in Fig. 3I-P, except that the number of stops and order was significantly lower in the BM1 than in the BM3 due to the shortened duration in the BM1. We omitted these results from the manuscript, because we focused on the differences of network structures between the BM1 and the BM3 given the same probe trial duration.

Eales, K. L., Palygin, O., O’Loughlin, T., Rasooli-Nejad, S., Gaestel, M., Müller, J., Collins, D. R., Pankratov, Y., & Corrêa, S. A. (2014). The MK2/3 cascade regulates AMPAR trafficking and cognitive flexibility. Nature communications, 5, 4701. https://doi-org.kyoto-u.idm.oclc.org/10.1038/ncomms5701

Comment #14

Fig. 5 seems better to be an Extended figure.

Reply

We have moved Figure 5 to Extended Data Figure 5-3.

Comment #15

The authors seem better to discuss about “route following” strategy of mice. There may be a possibility that Results of BM3 in Extended Figure 3 reflect it. In my point of view, the mice in BM3 seems to use “route following” strategy at first (by using external monitor cues), and then, acquire map-based navigation.

7

Reply

Please refer to our reply to Comment #4.
