# Supplementary figures and images for: Scale Space Calibrates Present and Subsequent Spatial Learning in Barnes Maze in Mice
Source: eNeuro. 2023 Jun 2;10(6):ENEURO.0505-22.2023. doi: 10.1523/ENEURO.0505-22.2023 (PMC10262649; doi:10.1523/ENEURO.0505-22.2023)

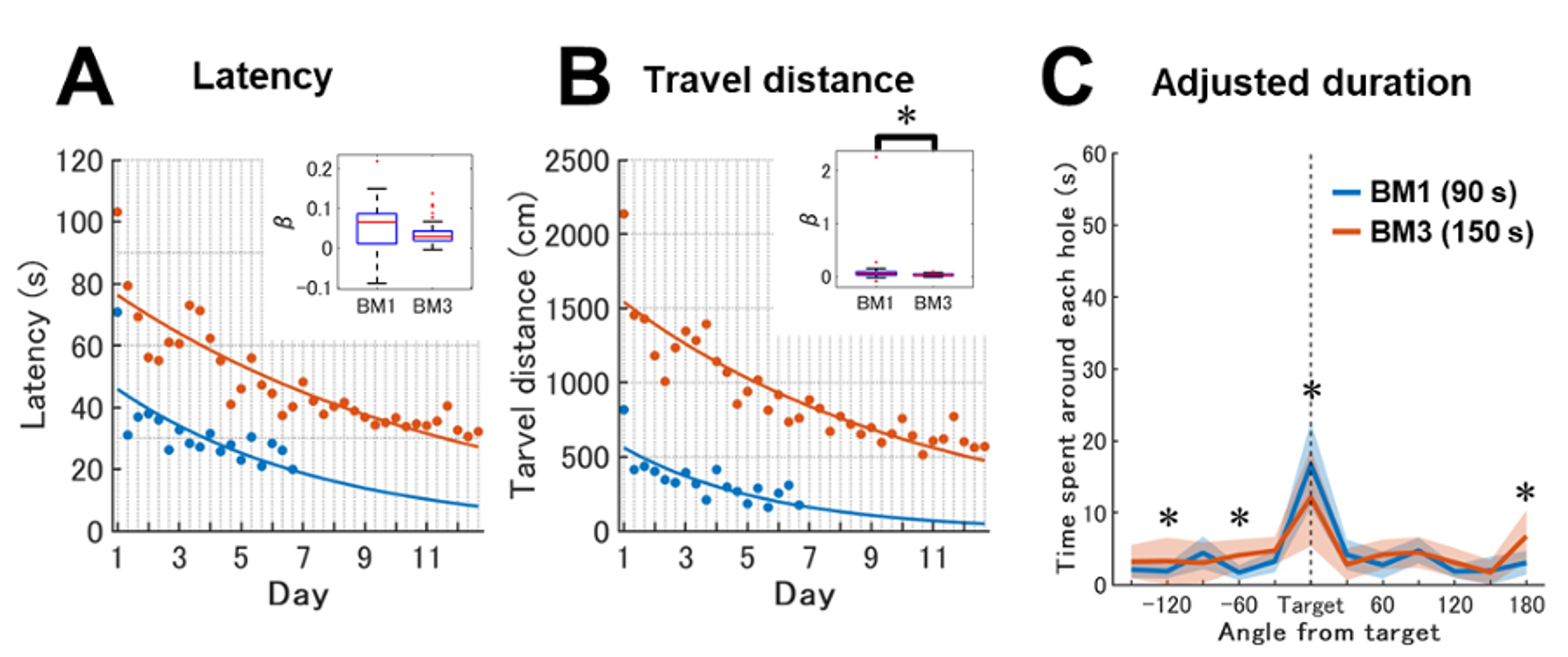

Supplement: Extended Data Figure 3-2 — Learning curves fitted for raw values of latency and travel distance and time spent around each hole given adjusted duration. While the BM1 and the BM3 would have different scale lower boundaries of latency and travel distance, respectively, while latency and travel distance should be compared between the BM1 and the BM3 within the same scale. So, latency and travel distance were normalized across trials so that the values range between 0 and 1 (Fig. 3B,C). A, A learning curve for raw values of latency. The vertical and horizontal axis indicates latency in second and training days, respectively. The same curve fitting method was used as Figure 3B. B, A learning curve for the raw values of travel distance. The vertical and horizontal axis indicates travel distance in centimeters and training days, respectively. The same curve fitting method was used as Figure 3C. The small inset in panels A and B is the distribution of estimated decay parameter β in the BM1 and the BM3. Asterisk indicates significant differences between the BM1 and the BM3. C, Time spent around each hole in the probe test under adjusted duration for the BM1 and the BM3. Duration for the BM1 and the BM3 was 90 s and 150 s, respectively, and was balanced so that the number of errors were statistically comparable between the BM1 and the BM3. Other than that, the graph format is identical to Figure 3H. Download Figure 3-2, TIF file. [file enu-eN-NWR-0505-22-s02.tif]

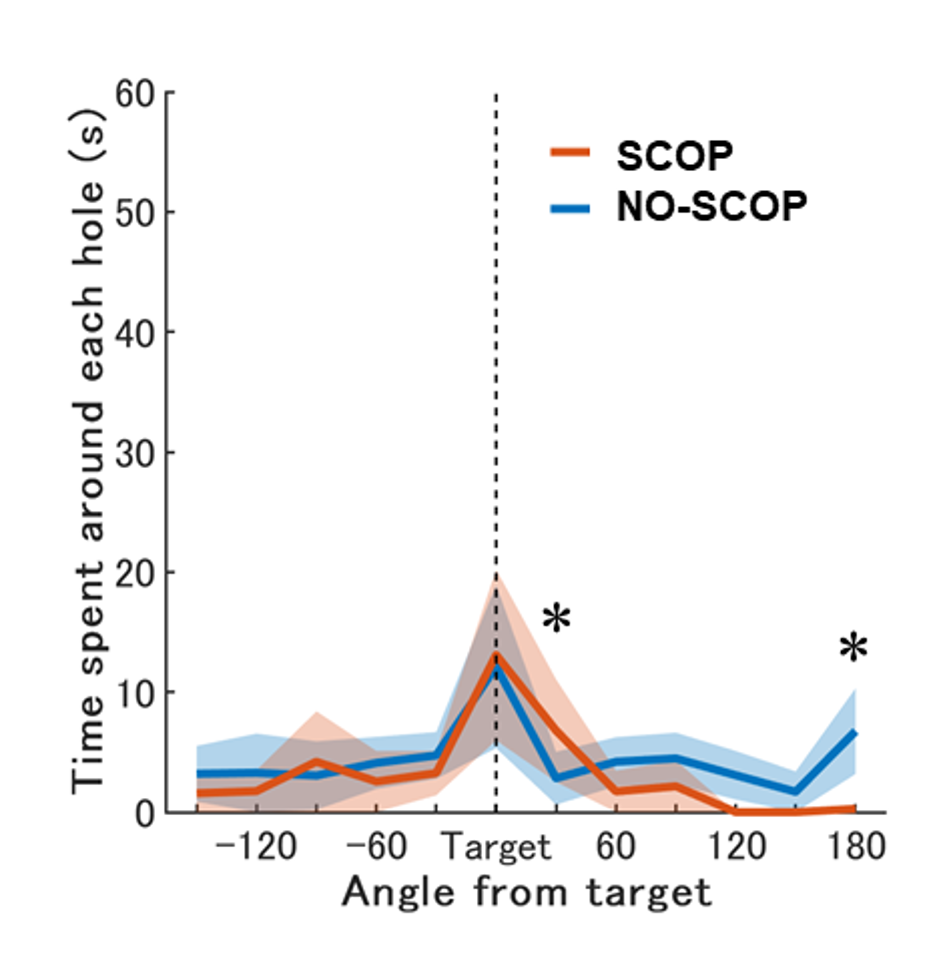

Supplement: Extended Data Figure 3-4 — Effects of scopolamine administration on the BM3 probe test. Time spent around each hole in the probe test. Asterisks indicate significant differences between the two groups. Red and blue represent the scopolamine-treated (SCOP) and untreated (NO-SCOP) mouse groups, respectively; the former is cohort S1 (n = 16), while the latter is the pool of Cohort 2 (n = 20) and the first instance of Cohort 3 (n = 20) in Table 1.. A mixed-design 2-way [Scopolamine (SCOP, NO-SCOP) × Hole (1∼12)] ANOVA for the time spent around each hole detected significant interaction between the scopolamine and hole, F(11,594) = 2.40, p = 0.01, ηp2 = 0.04. Multiple comparison testing detected that the time spent around target +30° was significantly longer in the SCOP than in the NO-SCOP whilst time spent around the opposite hole from the target was significantly shorter in the SCOP than in the NO-SCOP. Because a dummy escape tunnel was attached under the hole opposite to the goal hole (see Material and Methods), the NO-SCOP but not SCOP mice might search around it, once they observed that the true escape tunnel no longer exists at the goal hole. Download Figure 3-4, TIF file. [file enu-eN-NWR-0505-22-s03.tif]

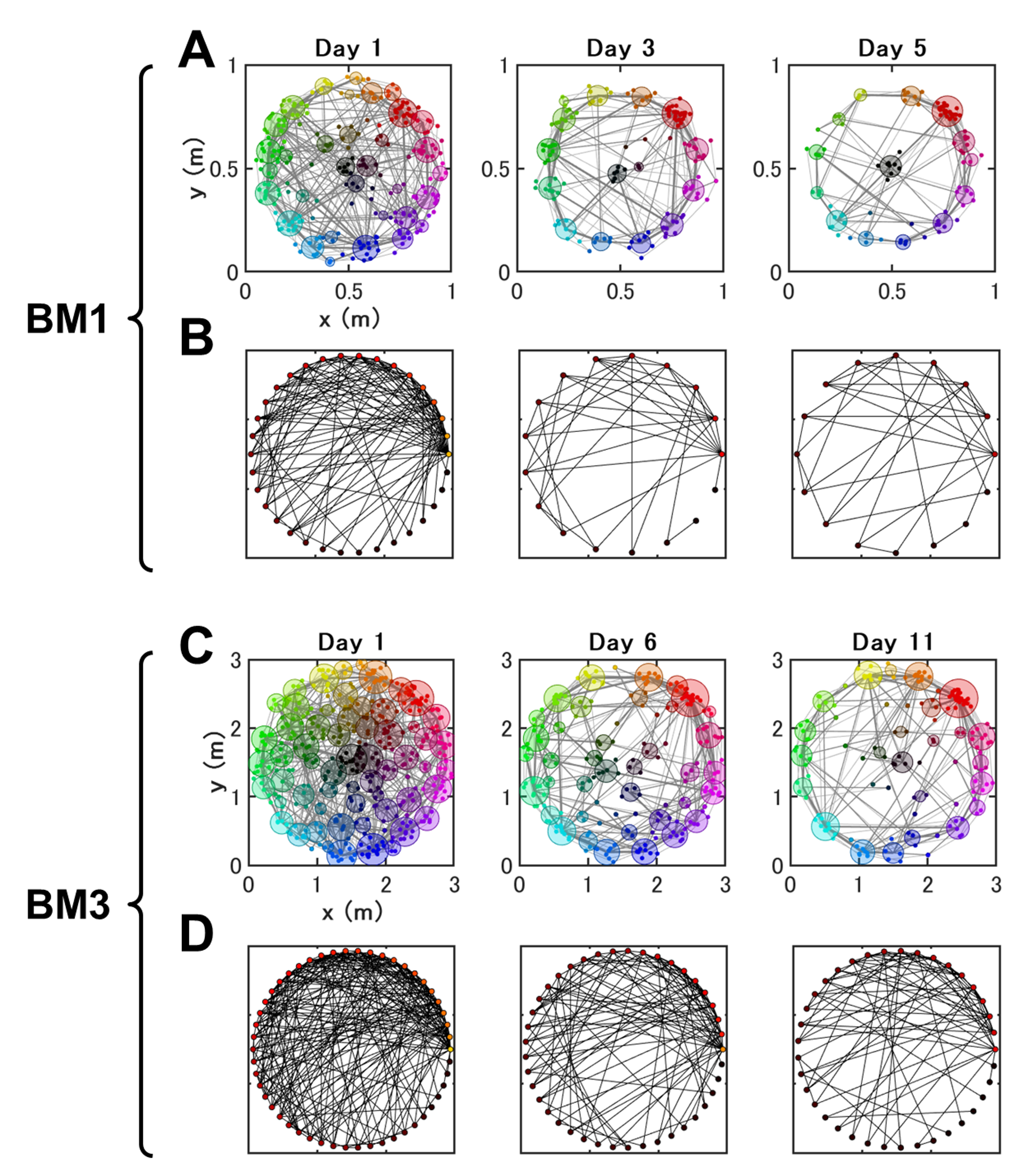

Supplement: Extended Data Figure 4-1 — Visualization of temporal changes in the exploration networks during spatial learning in the BM1 and BM3. A, Temporal changes in the global exploration networks in the BM1 spatial learning. Network structures of exploratory behaviors formed by the dynamic node generation method are plotted (Suzuki & Imayoshi, 2017). Small colored dots and light gray lines represent nodes and links in each mouse (local network). The nodes are located on Cartesian coordinates of the BM1. Nodes are color-coded depending on their polar coordinates. To visualize the global network structure, all local networks of the mice on a single training day were projected on a single plane. Colored larger circles and dark gray lines are global nodes and links in global networks, respectively. Local nodes belong to any one of the global nodes. Likewise, a set of local links are summarized as a global link. The size of a global node is based on log-transformation of the number of nodes that belong to the global node. Likewise, the thickness of a global link is log-transformation of the number of links that belong. B, Topological expression of BM1 global networks. Global nodes were sorted by rank-order of degree and plotted on polar coordinates, so that the node with the highest degree was located at 0 degrees while the lowest one was located at 360 degrees. Circles and lines represent global nodes and links, respectively. Global nodes were ranked to any one of 30 ranks depending on its degree within each group. Then, they were colored according to the rank; the rank first node has a lot of links and is colored by white, and rank 30th node has few links and is colored by black. C, D, Temporal changes in the global networks and their topological expressions of the BM3 spatial learning were displayed. Download Figure 4-1, TIF file. [file enu-eN-NWR-0505-22-s04.tif]

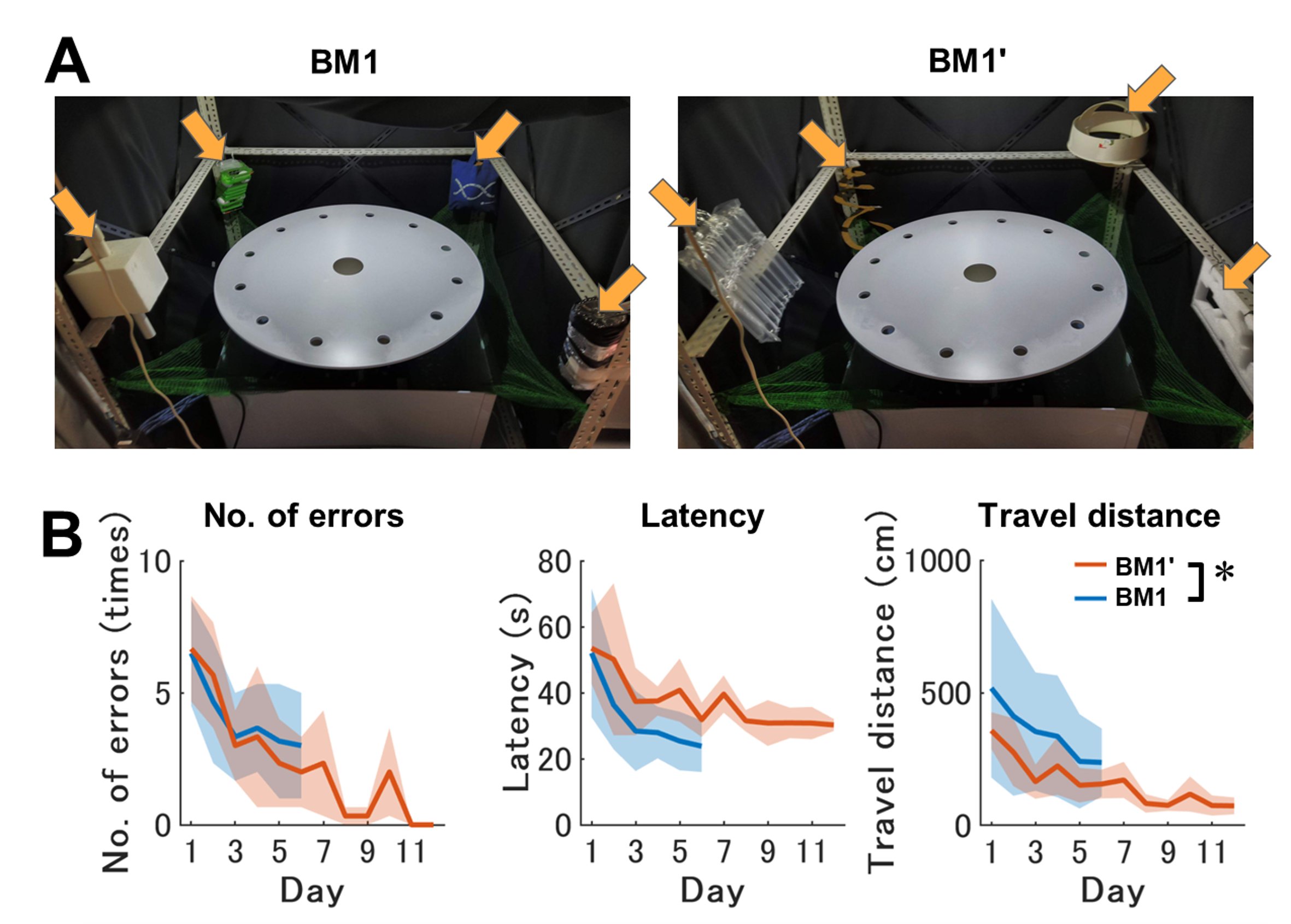

Supplement: Extended Data Figure 5-1 — The experiment setup and spatial learning curves in BM1 and BM1'. A, Spatial cues used in the BM1 (left panel) and the BM1' (right panel) were indicated with orange arrows. Note that all setups other than the cues were identical between the two mazes. B, Daily basis learning curves across training periods in conventional features in the BM1 and BM1'. These scores were averaged over 3 trials per day. From left, the measured values of number of errors, latency and travel distance are displayed. Only in a mixed-design 2-way [Spatial cues (BM1, BM1') × Day (1–6)] ANOVA for travel distance, the main effect of spatial cues was significant, F(1,49) = 6.72, p = 0.01, ηp2 = 0.12. Asterisks indicate significant differences between the two groups. Download Figure 5-1, TIF file. [file enu-eN-NWR-0505-22-s05.tif]

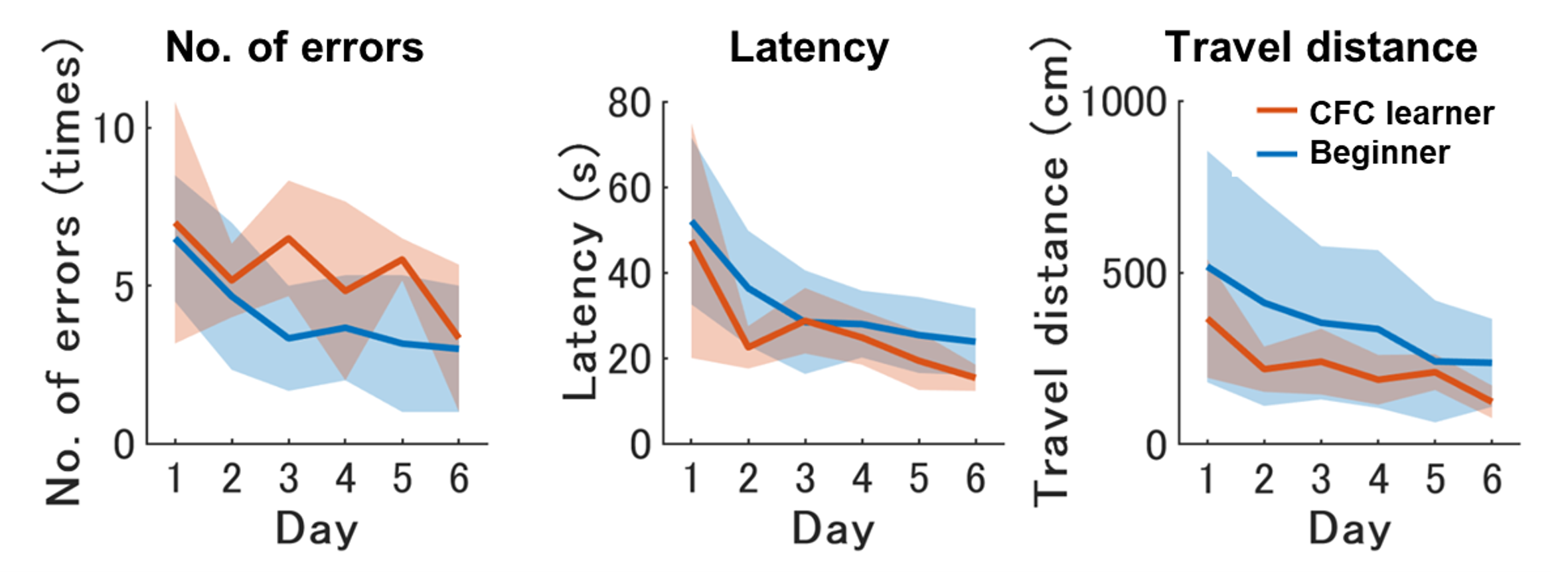

Supplement: Extended Data Figure 5-5 — Prior fear-conditioning experience does not impact on the subsequent BM1 spatial learning. Daily basis learning curves of the CFC learner n = 8) and Beginner (n = 34) across training periods in conventional features in the BM1. These scores were averaged over 3 trials per day. From left, the measured values of number of errors, latency and travel distance are displayed. Mixed-design 2-way [Instance (CFC learner, Beginner) × Day (1–6)] ANOVA detected neither main effect of Instance nor interaction between instance and day for all features. Download Figure 5-5, TIF file. [file enu-eN-NWR-0505-22-s06.tif]

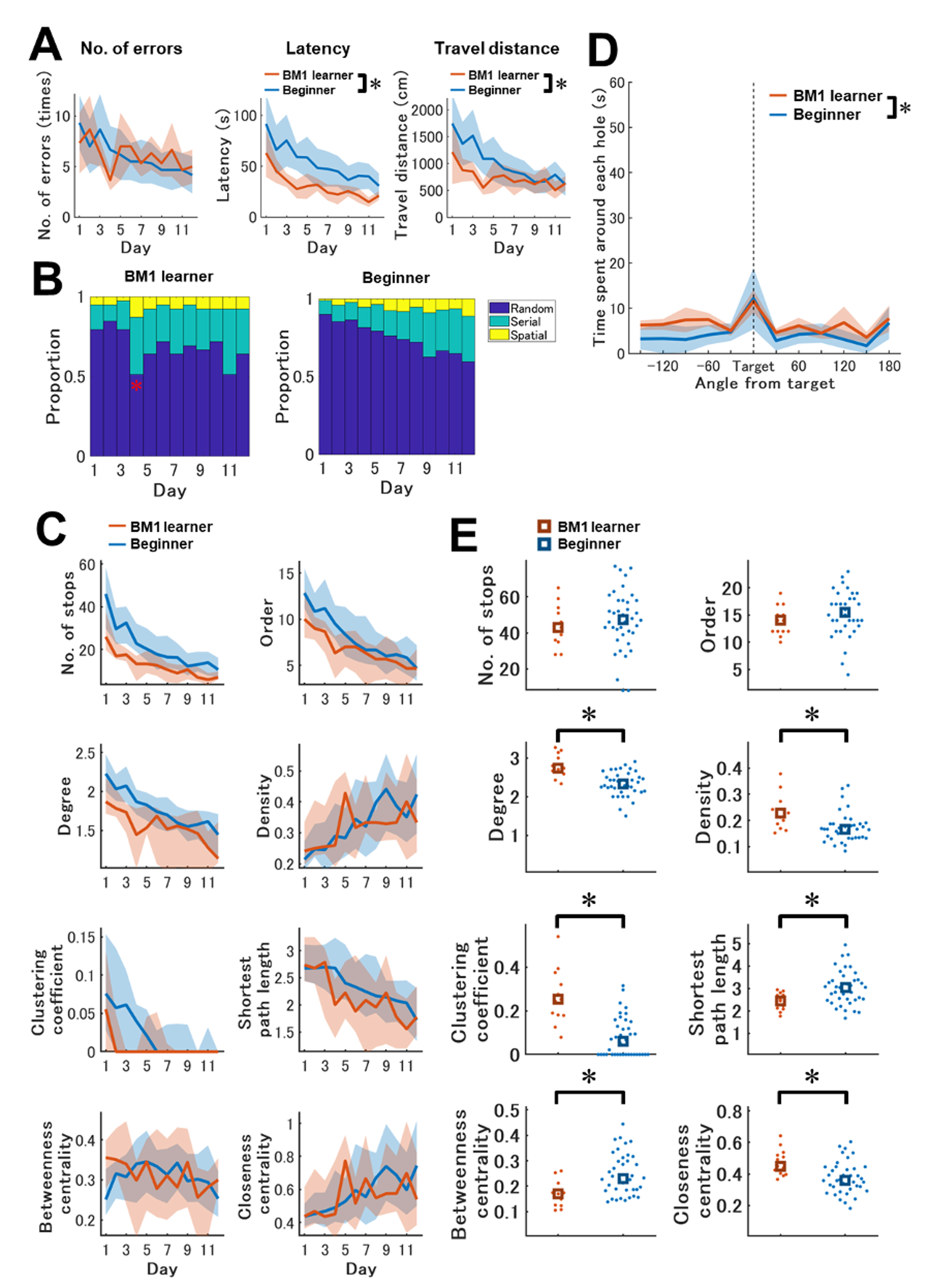

Supplement: Extended Data Figure 5-6 — Limited effects of spatial navigation and learning in the subsequent BM3 task by prior learnings in the BM1. A, Daily basis learning curves across training periods in conventional features in the BM3. These scores were averaged over 3 trials per day. From left, the measured values of number of errors, latency and travel distance are displayed. The mouse group of the BM1 learner (n = 13), which experienced the BM1 before the BM3, was compared with the Beginner (n = 56) group. Although the significant changes were detected in latency and travel distances, no significant difference was detected in the number of errors. B, Strategy usage across training days in the BM3 and comparison between BM1 learner and Beginner. Limited changes were detected only at the Day 4 results. C, Temporal changes of network features in the BM3 training of BM1 leaner and Beginner. Statistical results on each day are shown in Extended Data Figure 5-8. D, Time spent around each hole in the BM3 probe test. Only significant main effect of instance was observed; exploration time in the BM1 learner was significantly longer than that in the Beginner, regardless of hole location. E, Network features in the BM3 probe test compared between BM1 learner and Beginner. Asterisks indicate significant differences between the two groups. Download Figure 5-6, TIF file. [file enu-eN-NWR-0505-22-s07.tif]
