# Supplementary material for: Scale Space Calibrates Present and Subsequent Spatial Learning in Barnes Maze in Mice
Source: eNeuro. 2023 Jun 2;10(6):ENEURO.0505-22.2023. doi: 10.1523/ENEURO.0505-22.2023 (PMC10262649; doi:10.1523/ENEURO.0505-22.2023)
Supplement: Extended Data Figure 4-3 — Statistical results of network analysis for the BM1 and the BM3 in the BM1 probe test. Download Figure 4-3, DOCX file. [file enu-eN-NWR-0505-22-s11.docx]

# Extended Data Figure 4-3

Statistical results of network analysis for the BM1 and the BM3 in the BM1 probe test.

| Manuscript reference # | Figure | Measure | Comparison | Within | Data structure | Type of test | Statistic | p | Correction | ES |
| --- | --- | --- | --- | --- | --- | --- | --- | --- | --- | --- |
| 1 | Figure 4I | Number of stops | BM1 vs. BM3 | Probe test | No assumption | Wilcoxon rank-sum test | z = 0.81 | 0.42 | N/A | r = 0.09 |
| 2 | Figure 4J | Order | BM1 vs. BM3 | Probe test | No assumption | Wilcoxon rank-sum test | z = 0.86 | 0.39 | N/A | r = 0.10 |
| 3 | Figure 4K | Degree | BM1 vs. BM3 | Probe test | No assumption | Wilcoxon rank-sum test | z = 6.03 | 0.00* | N/A | r = 0.70 |
| 4 | Figure 4L | Density | BM1 vs. BM3 | Probe test | No assumption | Wilcoxon rank-sum test | z = 2.51 | 0.01* | N/A | r = 0.29 |
| 5 | Figure 4M | Clustering coefficient | BM1 vs. BM3 | Probe test | No assumption | Wilcoxon rank-sum test | z = 3.93 | 0.00* | N/A | r = 0.46 |
| 6 | Figure 4N | Shortest path length | BM1 vs. BM3 | Probe test | No assumption | Wilcoxon rank-sum test | z = -3.36 | 0.00* | N/A | r = -0.39 |
| 7 | Figure 4O | Betweenness centrality | BM1 vs. BM3 | Probe test | No assumption | Wilcoxon rank-sum test | z = -5.61 | 0.00* | N/A | r = -0.65 |
| 8 | Figure 4P | Closeness centrality | BM1 vs. BM3 | Probe test | No assumption | Wilcoxon rank-sum test | z = 3.47 | 0.00* | N/A | r = 0.40 |

Note. Asterisks indicate statistically significant differences. N/A: not applicable. ES: effect size.
